# Shape-Controlled Pathways in the Hydrogen Production from Ethanol Steam Reforming over Ceria Nanoparticles

**DOI:** 10.1021/acscatal.2c02117

**Published:** 2022-08-10

**Authors:** Julia Vecchietti, Patricia Pérez-Bailac, Pablo G. Lustemberg, Esteban L. Fornero, Laura Pascual, Marta V. Bosco, Arturo Martínez-Arias, M. Verónica Ganduglia-Pirovano, Adrian L. Bonivardi

**Affiliations:** † Instituto de Desarrollo Tecnológico para la Industria Química, UNL-CONICET, Güemes 3450, 3000 Santa Fe, Argentina; ‡ Instituto de Catálisis y Petroleoquímica, CSIC, C/Marie Curie 2, 28049 Madrid, Spain; § PhD Programme in Applied Chemistry, Doctoral School, Universidad Autónoma de Madrid, C/Francisco Tomas y Valiente 2, 28049 Madrid, Spain; ∥ Instituto de Física Rosario (IFIR), CONICET-UNR, Bv. 27 de Febrero 210bis, 2000EZP Rosario, Santa Fe, Argentina; ⊥ Facultad de Ingeniería Química, Universidad Nacional del Litoral, Santiago del Estero 2829, 3000 Santa Fe, Argentina

**Keywords:** ceria nanostructures, green hydrogen, ethanol decomposition, ethylenedioxy species, facet-dependent activity

## Abstract

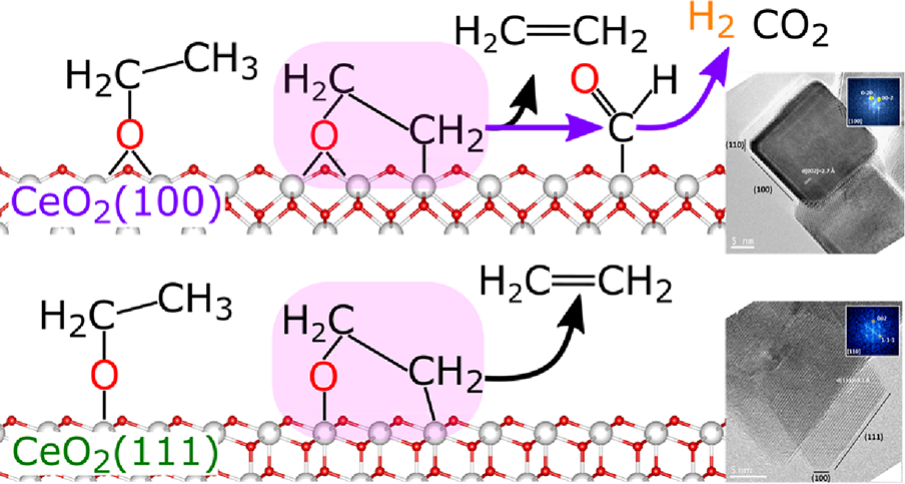

The ethanol surface reaction over CeO_2_ nanooctahedra (NO) and nanocubes (NC), which mainly expose (111) and (100) surfaces, respectively, was studied by means of infrared spectroscopy (TPSR-IR), mass spectrometry (TPSR-MS), and density functional theory (DFT) calculations. TPSR-MS results show that the production of H_2_ is 2.4 times higher on CeO_2_-NC than on CeO_2_-NO, which is rationalized starting from the different types of adsorbed ethoxy species controlled by the shape of the ceria particles. Over the CeO_2_(111) surface, monodentate type I and II ethoxy species with the alkyl chain perpendicular or parallel to the surface, respectively, were identified. Meanwhile, on the CeO_2_(100) surface, bidentate and monodentate type III ethoxy species on the checkerboard O-terminated surface and on a pyramid of the reconstructed (100) surface, respectively, are found. The more labile surface ethoxy species on each ceria nanoshape, which are the monodentate type I or III ethoxy on CeO_2_-NO and CeO_2_-NC, respectively, react on the surface to give acetate species that decompose to CO_2_ and CH_4_, while H_2_ is formed via the recombination of hydroxyl species. In addition, the more stable monodentate type II and bidentate ethoxy species on CeO_2_-NO and CeO_2_-NC, respectively, give an ethylenedioxy intermediate, the binding of which is facet-dependent. On the (111) facet, the less strongly bound ethylenedioxy desorbs as ethylene, whereas on the (100) facet, the more strongly bound intermediate also produces CO_2_ and H_2_ via formate species. Thus, on the (100) facet, an additional pathway toward H_2_ formation is found. ESR activity measurements show an enhanced H_2_ production on the nanocubes.

## 1 Introduction

The ethanol steam reforming reaction (ESR) represents an excellent opportunity to test the advantage of nanoshaped ceria catalysts to correlate the catalytic performance with the structure of the catalysts, while the increasing demand for clean and renewable energy sources has attracted attention toward the production of “green hydrogen”. Structure–activity relationships are essential toward the rational design of catalysts with improved activity and selectivity. The ESR reaction has the advantage of producing more H_2_ per mole of ethanol, compared to other catalytic routes.^1^ Additionally, ethanol has become an important energy vector due to its non-toxicity, easy storage, and safe handling and the fact that it can be obtained from renewable resources.^2^ One of the main challenges with respect to the formulation of catalytic materials for the ESR reaction lies in the development of active metal-supported catalysts with enhanced yield toward H_2_ and inhibition of undesired byproducts that can lead to coke formation.^3−6^

The use of ″real″ catalysts (for example, polycrystalline powders) makes the unequivocal correlation between the surface structure of the catalyst and the catalytic performance a very difficult task due to the inhomogeneity of the particles in their sizes and morphologies. In this context, the strategy of using model catalysts that capture part of the complexity of the real systems emerges as an alternative to establishing well-defined relationships between structure and catalytic function.^7^ Traditional model catalysts are based primarily on single crystals and the studies are generally performed under ultrahigh vacuum (UHV) conditions. These model catalysts are very useful for addressing the effect of the surface exposed crystallographic planes on catalytic performance.^8−10^ However, the so-called ″material gap″ and ″pressure gap″ between those model single crystal-based catalysts and the corresponding powder catalysts exist.^11,12^ Model nanocrystal-based powder catalysts with well-defined shapes that expose specific facets provide a means to bridge these gaps. To that end, various types of nanostructured materials (oxides) with a uniform composition and structure (size and morphology) can be successfully synthesized.^13−16^ These materials constitute a new type of model catalysts to explore relationships between structure and catalytic performance.

In particular, it has been reported that cerium oxide can be suitable as a support for ESR metal-oxide catalysts due to its high oxygen storage capacity and at the same time that oxygen mobility can improve catalytic stability avoiding sintering of metal particles and suppressing formation of carbonaceous species.^17^ Thus, using the hydrothermal method, CeO_2_ nanostructures are obtained with preferential exposure of certain crystallographic planes: (111) for nanooctahedra, (100) for nanocubes, and a mixture of (110) and (100) facets for nanorods,^18−21^ which presented different catalytic activity for some catalytic processes,^22−27^ but reports on the ESR reaction are scarce.^24,28−35^ Soykal et al. studied Co catalysts supported on ceria nanorods and nanocubes. The activity measurements over the bare supports showed that ceria nanocubes were more active and showed better C–C cleavage activity compared to the rods.^24^ The Co catalysts supported on the cubes showed higher H_2_ and CO_2_ yields, whereas Co/CeO_2_-nanorods were only active for dehydrogenation and dehydration. The superior performance of Co/CeO_2_ nanocubes catalysts was thought to be due to a combination of factors, including improved metal dispersion, increased reducibility, and higher oxygen mobility. Moraes and collaborators studied the effects of ceria morphology (nanocubes, nanorods, and flower-like) on the catalytic performance of Ni/CeO_2_ catalysts for the ESR reaction.^28^ The ESR activity measurements, performed at 300 °C over pre-reduced catalysts, showed that the product distribution was not affected by the ceria morphology and that the ethanol decomposition and dehydrogenation were the main reactions for all catalysts. Another work by Araiza et al. focused on the effect of ceria nanostructures (particles, rods, and cubes) on the carbon deposition during steam reforming of ethanol over 10% Ni/CeO_2_ catalysts.^34^ Nickel supported on ceria nanorods exhibited the best activity and hydrogen yield in the ESR reaction at 550 °C for 24 h and presented the lower amount of carbon deposits. These characteristics of the rod-shaped catalyst were ascribed to the enhanced oxygen storage capacity presented by ceria rods and the higher dispersion of nickel over this last ceria nanoshape. Most recently, Kourtelesis et al. studied the ethanol steam reforming over Pt/CeO_2_ with different support morphologies (cubes, rods, and flower-like).^29^ The support morphology was found to influence certain reaction routes, even though the reaction scheme was the same regardless of the support. Pt supported on ceria nanocubes exhibited the highest initial ethanol conversion.

All of these works have mainly focused on the support morphology effect on the catalytic activity of a catalyst with the metallic function on the surface. Concerning pure ceria, Li et al. have studied the temperature programmed desorption (TPD) of ethanol by mass spectrometry (MS) and infrared spectroscopy (IR) over CeO_2_ nanorods, nanocubes, and nanooctahedra.^22^ However, this work focused on the catalytic oxidation of ethanol rather than the understanding of ethanol chemisorption and decomposition on the different ceria nanoshapes. Therefore, to the best of our knowledge, a comprehensive study on the adsorption and decomposition of ethanol on the surface of differently shaped ceria nanoparticles that combine experimental work and DFT calculations is missing.

Thus, in this work, the interaction of ethanol with the surface of CeO_2_ nanocubes and nanooctahedra, which mainly expose the (100) and (111) facets on the surface, is studied employing temperature-programmed surface reaction (TPSR) of ethanol by means of infrared spectroscopy (IR) and mass spectrometry (MS) combined with DFT calculations, and the facet-dependent chemisorption and decomposition properties are discussed. Moreover, the catalytic performance of these different ceria nanoshapes is evaluated for the ESR reaction. We show that the modification of the shape, surface/face reconstruction of ceria crystallites at the nanoscale, can offer an important tool to control activity and selectivity in the ESR reaction. In particular, the role of ethylenedioxy species, OCH_2_CH_2_O_latt_, in enhancing H_2_ formation has been compellingly shown, shining a light on the discrepancy between results on extended ceria surfaces and on ceria nanoshaped crystallites in the literature.

## 2 Experimental Section

### 2.1 Synthesis of Materials

The cerium oxide nanocubes (CeO_2_-NC) were synthesized by a hydrothermal method.^20^ Ce(NO_3_)_3_·6H_2_O (99.99%, Sigma-Aldrich) and NaOH (Merck, 99%) were used for the preparation of the nanocubes. Appropriate amounts of Ce(NO_3_)_3_·6H_2_O (115 mL, 0.1 M) and NaOH solutions (125 mL, 11.5 M) were mixed and stirred in a Teflon 300 mL vessel for 30 min. Then, the Teflon reactor was introduced in a stainless steel autoclave and heated at 180 °C for 24 h in an electric oven. After the hydrothermal synthesis, the mixture was cooled down to room temperature, and the precipitate was separated from the aqueous solution by centrifugation. The solid was washed several times with water followed by ethanol and then dried in an oven at 60 °C for 24 h. The dried material was calcined at 450 °C for 5 h (2 °C/min) in a glass tubular reactor under flowing 20% O_2_/N_2_ mixture (5 mL/min/g).

The synthesis of CeO_2_ nanooctahedra (CeO_2_-NO) was performed also by a hydrothermal method but using an approach similar to that of Han et al.^21^ An important characteristic of the method employed has been to avoid the use of a phosphate salt as the precursor, as done in most of the recipes employed for preparation of this particular type of nanoshapes since the first works dedicated to this topic.^19^ Note that an important concern in this sense is that, as demonstrated by Wu et al.,^36,37^ phosphate is difficult to eliminate from the sample surface during the rinsing steps involved in the preparation and its presence, even at very low concentration, importantly modifies the surface acid–base or redox properties of CeO_2_.^38^ Thus, for that purpose, 1 g of Ce(NO_3_)_3_·6H_2_O was diluted in 10 mL of deionized water and added dropwise to 50 mL of a 0.01 M solution of NaOH. After stirring for at least 15 min, water was added to obtain a final volume of 80 mL (pH ∼ 7). Then, the mixture was introduced in an autoclave and heated for 24 h at 180 °C. At the end of the autothermal heating, the mixture was cooled to room temperature (pH ∼ 2); then, the solid was separated by centrifugation and washed several times with water followed by ethanol. Finally, the sample was dried at 80 °C for 12 h and calcined under air at 500 °C for 2 h (2 °C/min).

### 2.2 Characterization

The Brunauer–Emmett–Teller surface area (S_BET_) of each material, previously outgassed at 200 °C for 2 h under dynamic vacuum (base pressure = 1.339 10^–4^ Pa), was measured at −196 °C (LN2) using Micromeritics ASAP-2020 apparatus.

Transmission electron microscopy (TEM) was performed with a field emission gun TEM/STEM (JEOL 2100 F) operating at 200 kV, providing a point resolution of 0.19 nm.

### 2.3 Temperature-Programmed Surface Reaction of Ethanol

Two kinds of temperature-programmed surface reaction (TPSR) studies of adsorbed ethanol were carried out to follow the adsorbed and gaseous-phase evolved species, by infrared (IR) and mass spectrometry (MS), respectively, as follows:

#### 2.3.1 TPSR by Infrared Fourier Transform Spectroscopy (TPSR-IR)

TPSR-IR experiments were performed by in situ transmission IR spectroscopy using a Nicolet 8700 FTIR spectrometer operated with a Hg-Cd-Te detector. Self-supported wafers of each solid (approximately 30 mg/cm^2^) were placed in an electrically heated glass flow-through cell fitted with NaCl windows. Before the alcohol adsorption, the samples were pretreated for carbonate surface removal as follows: (1) heating under pure H_2_ flow to 450 °C (15 min); (2) evacuation at 450 °C (15 min); (3) oxidation with O_2_ at 450 °C (15 min); (4) cooling under O_2_ flow to 100 °C; and (5) evacuation at 100 °C (15 min). Then, a pulse of gaseous ethanol (500 μmol) was admitted into the cell and afterward evacuated to eliminate the ethanol excess (15 min, 100 °C). Finally, the cell was heated until 450 °C (5 °C/min) under He flow. In all cases, the gas flow was 50 cm^3^/min. The IR spectra were taken consecutively by averaging 25 scans (acquisition time = 30 s) and with a resolution equal to 4 cm^–1^.

#### 2.3.2 TPSR by Mass Spectrometry (TPSR-MS)

A sample amount equivalent to 10 m^2^ of each oxide, diluted with quartz, was loaded on a U shaped microreactor coupled with a mass spectrometer (Balzers QMG 112A). The same cleaning treatment protocol was implemented, as detailed in the previous TPSR-IR experiment, but purging was done under He flow (instead of vacuum). Ethanol adsorption was performed by injecting ethanol pulses of 10 μL into an evaporator at 250 °C, until the area of ethanol pulses remained unchanged. Afterward, TPSR was performed by heating the reactor until 450 °C (5 °C/min) under He flow (50 cm^3^/min). During the TPSR-MS, the following species were scanned at the outlet of the reactor: H_2_, He, N_2_, CH_4_, H_2_O, C_2_H_4_ (ethylene), CO, C_2_H_4_O (acetaldehyde), C_2_H_5_OH (ethanol), O_2_, CO_2_, C_3_H_6_O (acetone), (C_2_H_5_)_2_O (diethyl ether), C_2_H_4_O_2_ (acetic acid), and C_4_H_8_O_2_ (ethyl acetate) (that is, *m*/*e* = 2, 4, 14, 16, 18, 27, 28, 29, 31, 32, 44, 58, 59, 60, and 61 amu, respectively). The amount of absorbed ethanol (ethanol uptake) was determined from the difference between the area of an ethanol pulse measured bypassing the reactor and the area of the pulse taken during the adsorption of ethanol.

### 2.4 Theoretical Models and Computational Methods

Density functional calculations (DFT) were carried out using the slab–supercell approach^39^ with the Vienna ab initio simulation package (VASP, http://www.vasp.at; vasp version 5.4.4).^40,41^ We explicitly treated the Ce (4f, 5s, 5p, 5d, 6s), O (2s, 2p), C (2s, 2p), and H (1s) electrons as valence states within the projector augmented wave (PAW) method with a plane-wave cutoff energy of 600 eV, whereas the remaining electrons were considered as part of the atomic cores. Strong correlation effects due to charge localization were modeled by adding a Hubbard *U*-like term^42^ (*U*_eff_ = *U* – *J*, i.e., the difference between the Coulomb *U* and exchange *J* parameters, from now on referred to simply as *U*) to the Perdew, Burke, and Ernzerhof (PBE) generalized gradient approximation (GGA) functional.^43^ We used a value of *U* = 4.5 eV for the Ce 4f states.^44,45^ Long-range dispersion corrections were considered employing the so-called DFT-D3 approach.^46,47^ The adsorption energies and vibrational frequencies reported in this work for the case of the (111)-oriented CeO_2_ surface differ insignificantly from those reported by some of us in ref (48) due to the different description used for the long-range dispersion corrections.

**Figure 1:**
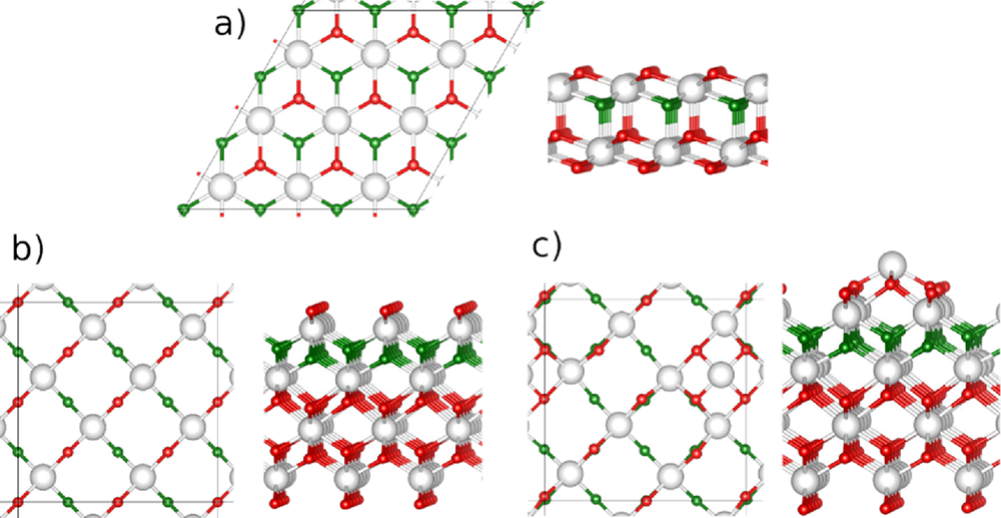
Top and side views of the clean ceria surfaces: (a) (3 × 3)-(111), (b) c(2 × 2)-(100) O-terminated, and (c) c(2 × 2)-(100) with a mixture of O and Ce terminations (75% [(100)-O]–25% [(100)-Ce]). Color code: Ce (O) atoms in the outermost layer are white (red/green). This color code is used in all subsequent figures.

The three ceria surface models (see Figure 1) used in the present work were created from the ceria bulk with a DFT-calculated lattice parameter of 5.485 Å. The (111) termination was modeled with 3 × 3 periodicity and a six atomic layer slab (two O–Ce–O trilayers). In an earlier work,^48^ selected calculations have been carried out with nine atomic layer slabs (three O–Ce–O trilayers) and the results indicated that the thickness of the slab model in the calculated properties such as the adsorption energy of dissociatively adsorbed ethanol species and their vibrational frequencies did not have a noticeable effect. In the case of the CeO_2_(100) surface, the checkerboard O termination and a mixture of the O and Ce terminations (75% [(100)-O]–25% [(100)-Ce]) with c(2 × 2) periodicity, as described previously by Pérez-Bailac et al.,^49^ hereinafter referred to as (100)-O and (100)-Mix, respectively, have been considered. The O and Ce terminations have only 50 % of the atoms in the surface layer, compared to corresponding deeper layers, and the (100)-Mix c(2 × 2) termination has a single nanopyramid with a topmost Ce ion and four anions in the layer below. The selection of these two terminations is due to the fact that the surface energy of the (100)-Mix termination (1.42 J/m^2^) is only 0.03 J/m^2^ smaller than that of the pure (100)-O termination (1.45 J/m^2^), whereas that of the Ce-terminated one is 0.32 J/m^2^ larger,^49^ in agreement with what was observed by Pan et al.^50^ During geometry optimizations, only the three bottom layers of the slabs have been kept fixed at their optimized bulk-truncated positions. The composition of the slabs was Ce_18_O_36_, Ce_32_O_64_, and Ce_33_O_66_ for the pure ceria (111), (100)-O, and (100)-Mix terminations, respectively.

In a previous work,^48^ the important influence of the presence of water and hydroxyl species on the CeO_2_(111) surface on the vibrational frequencies of ethoxy species was observed. It has been earlier reported that the dissociative adsorption of water is stronger on the (100) surface than on (111).^51,52^ The coordinative unsaturation of the (100) surface favors the dissociative adsorption of water and the full hydroxylation of the surface. Therefore, a higher degree of hydroxylation is expected on the (100) surface as compared to the (111). In line with those results, our models for the hydroxylated ceria surfaces have been constructed with one dissociated water molecule on the (111) surface and three on the (100)-O and (100)-Mix surfaces, resulting in slabs with Ce_18_O_37_H_2_, Ce_32_O_67_H_6_, and Ce_33_O_69_H_6_ compositions, respectively. We point out that in this work, the most stable configuration of dissociated water and ethanol has not been sought, but rather how the presence of neighboring hydroxyl groups influences the nature and stability of adsorbed ethoxy species and their vibrational frequencies. We further note that the catalytic activity of the clean and hydroxylated (100)-Mix surfaces has been considered in several recent studies.^53,54^

The adsorption energy of the dissociatively chemisorbed ethanol was calculated according to the following equation for the hydroxylated ceria surfaces: *E*_ads_ = *E*[(C_2_H_5_O + H)/(hydro-ceria)] – *E*[hydro-ceria] – *E*[C_2_H_5_OH_gas_], where *E*[(C_2_H_5_O + H)/(hydro-ceria)] is the total energy of the ethoxy species and hydrogen co-adsorbed on the hydroxylated surface, *E*[hydro-ceria] is the total energy of the surface where C_2_H_5_O + H adsorbates were removed and structures optimized (Figure S1), and *E*[C_2_H_5_OH_gas_] is the energy of the gas phase ethanol molecule.

The frequencies and intensities have been calculated using the density functional perturbation theory (DFPT).^55−58^ Within the dipolar approximation, the intensity of the infrared active modes may be calculated^59^ in terms of the oscillator strengths^60^ determined by the Born effective charges and the displacement vectors as described by Karhánek et al.^61^ The frequencies have been scaled, following the procedure described by Merrick et al.^62^ in the ranges from 800 to 1200 and from 2800 and 3200 cm^–1^ (see the Supporting Information and Table S1), obtaining a scale factor of λ = 1.019 and λ = 0.987 in each range, respectively.

### 2.5 Catalytic Test

Each sample was diluted with quartz (1:4 w/w), placed in a quartz microreactor, and then subjected to the same cleaning pretreatment as detailed previously for the TPSR experiments. The catalytic test was run with a mixture of water and ethanol (H_2_O:C_2_H_5_OH = 6:1 mol-to-mol) diluted in Ar (H_2_O:C_2_H_5_OH:Ar = 8.8:1.5:89.7 molar ratio). The catalytic performance was measured from 300 to 450 °C (50 °C steps, 1 h at each temperature) using two gas chromatographs Shimadzu GC-9A equipped with a Porapack QS and Carbosieve SII and TCD and FID detectors for the quantification of C_2_H_5_OH, H_2_, CO, CO_2_, CH_4_, C_2_H_4_ (ethylene), C_2_H_4_O (acetaldehyde), CH_3_COCH_3_ (acetone), and CH_3_CHOHCH_3_ (2-propanol). The residence time was normalized per surface area of the oxides (*S*_CeO_2__/*F*°_C_2_H_5_OH_ = 800 m^2^ h/mol_C_2_H_5_OH_, where *S*_CeO_2__ stands for the surface area of the sample and *F*°_C_2_H_5_OH_ stands for the molar flow of ethanol at the inlet of the reactor). Conversion of ethanol (*X*_C_2_H_5_OH_) and yield to carbon-containing compounds (Yield_i_) were calculated (for details, see the Supporting Information).

In order to determine the apparent activation energies (*E*_a_) for ethylene and acetone production, additional experiments were performed under differential ethanol conversion conditions (lower than 10%): H_2_O:C_2_H_5_OH = 6:1 mol-to-mol; F°_tot_ = 300 cm^3^/min (where F°_tot_ stands for the total molar flow at the inlet of the reactor); *T* = 380–430 °C; *S*_CeO_2__/*F*°_C_2_H_5_OH_ = 274 and 530 m^2^ h/mol_C_2_H_5_OH_ for CeO_2_-NO and CeO_2_-NC, respectively. Furthermore, to better compare the values of selectivity of both materials under iso-conversion and isothermal conditions (*X*_C_2_H_5_OH_ ∼ 5% at 400 °C), the space velocity employed with CeO_2_-NO was adjusted accordingly (*F*°_tot_ = 415 cm^3^/min corresponding to *S*_CeO_2__/*F*°_C_2_H_5_OH_ = 212 m^2^ h/mol_C_2_H_5_OH_).

## 3 Results

### 3.1 Characterization

**Figure 2:**
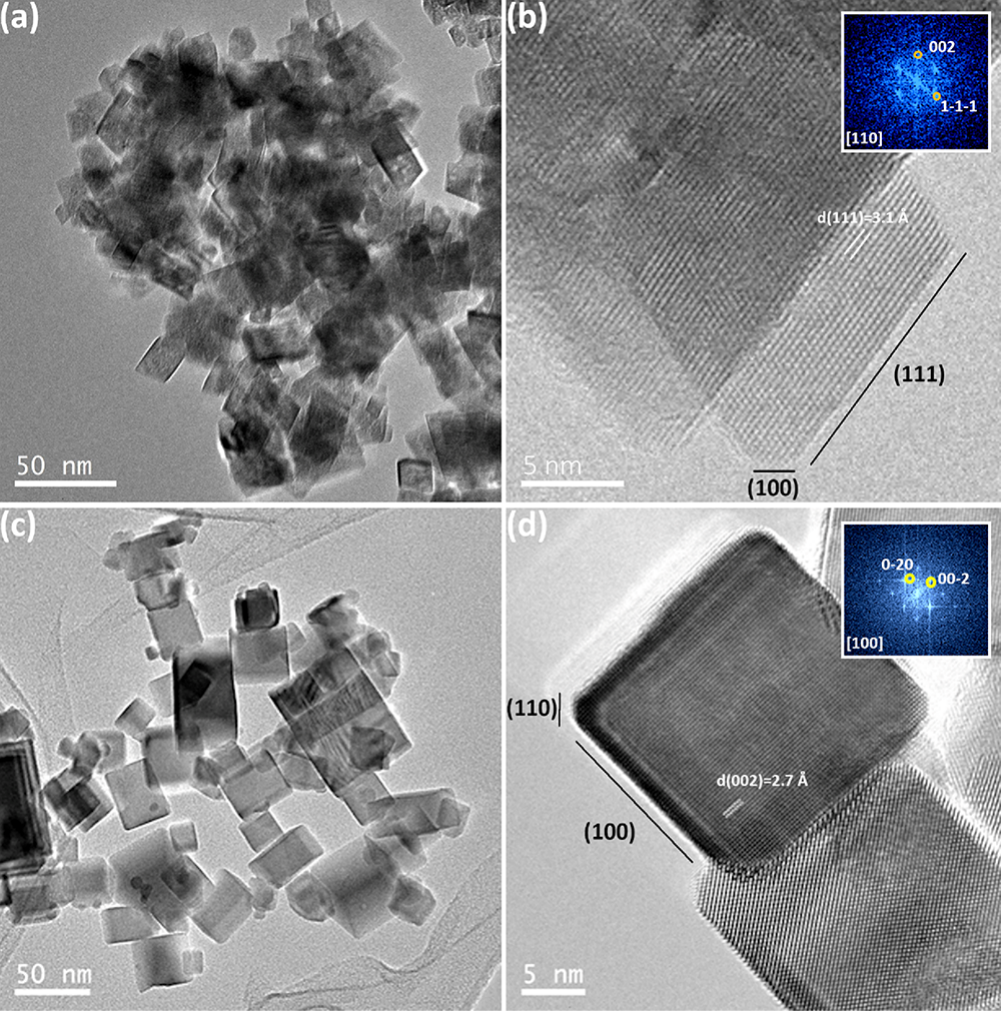
TEM and HREM images for CeO_2_-NO (a,b) and CeO_2_-NC (c,d). Inset in panels (b) and (d) are the FFT of that image.

The morphology and microstructure of both ceria samples were studied by transmission electron microscopy. The low-magnification TEM micrograph in Figure 2a shows the presence of aggregates of octahedra-shaped nanoparticles (CeO_2_-NO) with a size ranging from 10 to 40 nm and an average particle size of 23.2 nm. A more detailed high-resolution HRTEM analysis of a faceted octahedra particle is shown in Figure 2b, which is perfectly oriented along the [110] zone axis of the fluorite type structure, as can also be deduced from the FFT (fast Fourier transform) shown in the inset. Moreover, it can be observed that the octahedral particles are not perfectly shaped because they present truncated vertices. This type of truncation exposes (100) facets and is in good agreement with the results previously described in the literature.^63^

Figure 2c shows a representative TEM micrograph of the CeO_2_-NC sample. In this case, a cube-shaped morphology of the nanoparticles is displayed. The particle sizes vary within a 10 to 50 nm range, with an average particle size of 30 nm. The HRTEM micrograph (Figure 2d) reveals the microstructure of a cube, which is oriented along the [100] zone axis of the fluorite type structure (see the FFT in the inset) where slight truncations exposing (110) facets are observed.

As expected from the average particle sizes, the BET surface area was higher for CeO_2_-NO than for CeO_2_-NC, that is, 57 vs 28 m^2^/g, respectively (see Table 1).

**Table 1:** Characterization of the CeO_2_ Nanoshapes

sample	*S*_BET_ (m^2^/g)^a^	average size (nm)^b^	ethanol uptake (μmol C_2_H_5_OH/m^2^)^c^
CeO_2_-NO	57	23.2	4.2 ± 0.2
CeO_2_-NC	28	30.0	3.8 ± 0.2

a Brunauer–Emmett–Teller (BET) surface area.

b Determined by TEM.

c Mass Spectrometry (MS) at 100 °C (see text).

### 3.2 Adsorption and Decomposition of Ethanol over Ceria Nanooctahedra and Nanocubes

#### 3.2.1 Temperature-Programed Surface Reaction by Mass Spectrometry (TPSR-MS)

**Figure 3:**
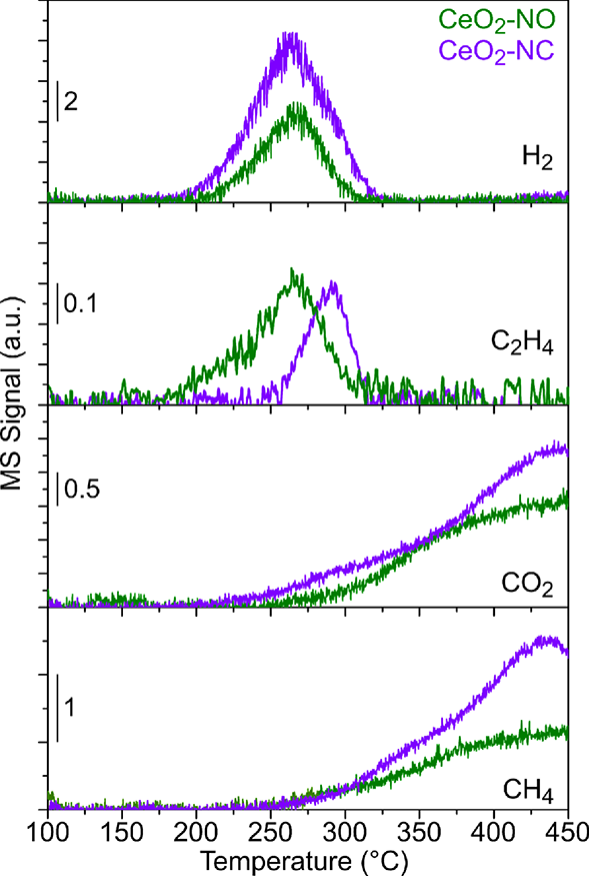
Traces of H_2_, CO_2_, CH_4_, and C_2_H_4_ obtained during the TPSR-MS for CeO_2_-NO and CeO_2_-NC samples (10 m^2^ of each sample was loaded into the reactor).

The ethanol temperature-programmed surface reaction was studied by mass spectrometry (TPSR-MS). The ethanol uptakes calculated after adsorption over the octahedra- and cube-shaped nanoparticles (see Section 2.3.2) are reported on Table 1. The amount of ethanol adsorbed on both nanoshapes studied in this work was approximately 4 μmol EtOH/m^2^. Figure 3 shows the main gaseous products detected at the outlet of the reactor for CeO_2_-NO and CeO_2_-NC. The temperature-programmed surface reaction of ethanol produces H_2_, C_2_H_4_, CO_2_, and CH_4_ over the surfaces of both ceria nanooctahedra and nanocubes, which mainly expose (111) and (100) CeO_2_ facets, respectively, as described above. However, some differences can be observed.

On CeO_2_-NO, H_2_ begins to be detected at 200 °C, reaching a maximum at 268 °C. Then, the intensity of the molecular hydrogen MS signal begins to decrease, disappearing completely at 310 °C. Ethylene has a similar evolution as H_2_; however, it is detected at lower temperatures (175 °C) with a shoulder at 210 °C. CO_2_ evolves together with CH_4_; thus, both products start to be measurable at 250 °C and their intensities increase up to 450 °C.

On the other hand, H_2_ production on CeO_2_-NC onsets at lower temperatures (175 °C) than on nanooctahedra, with a maximum at approximately the same temperature (264 °C), but the integrated area of the H_2_ evolution is 2.4 higher on the nanocubes. Ethylene, however, is detected above 240 °C reaching a maximum at 290 °C. In other words, in the case of the nanocubes, the development of the H_2_ and ethylene signals occurs at different temperatures. CeO_2_-NC produces ethylene at higher temperature compared to CeO_2_-NO, and there is not a low temperature shoulder as in the case of the nanooctahedra. Additionally, the integrated area of the ethylene peak is approximately half smaller in ceria cubes than in octahedra. CO_2_ and CH_4_ are also released after ethanol adsorption and decomposition on the CeO_2_-NC: both gases are detected from 250 °C, reaching a maximum at 437 °C. The amount of CO_2_ and CH_4_ released in the gas phase is 1.4 and 1.7 times larger, respectively, for the cubes than for the octahedra.

From our TPSR-MS experiments, it can be concluded that the underlying chemistry of both ceria nanoshapes is similar since the same type of species is produced in the gas phase after adsorption and decomposition of ethanol. However, the differences observed between the cubes and the octahedra with respect to the amounts and evolution temperature of the gaseous products are worth to be further analyzed. Thus, to better understand the decomposition of ethanol on ceria surfaces and to find correlations between the evolution of gaseous products and that of adsorbed surface species, we have also studied the nature of the latter by means of IR spectroscopy.

#### 3.2.2 Ethanol Adsorption on CeO_2_ Nanoshapes by IR and DFT

**Figure 4:**
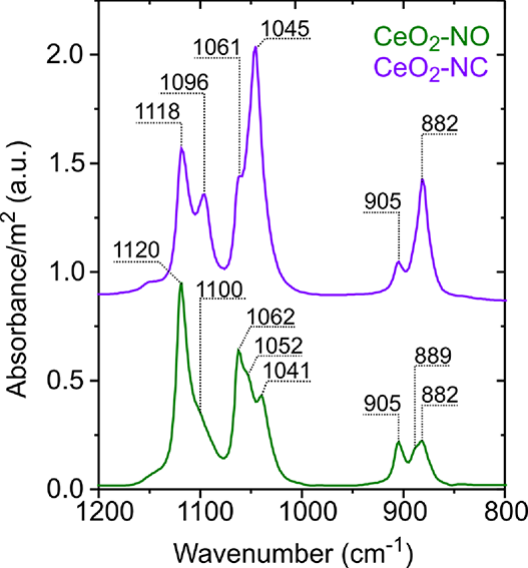
Normalized IR spectra for CeO_2_-NO (nanooctahedra) and CeO_2_-NC (nanocubes) after ethanol adsorption at 100 °C and purging with He. The spectrum of the clean oxide right before the adsorption was subtracted.

**Scheme 1:**
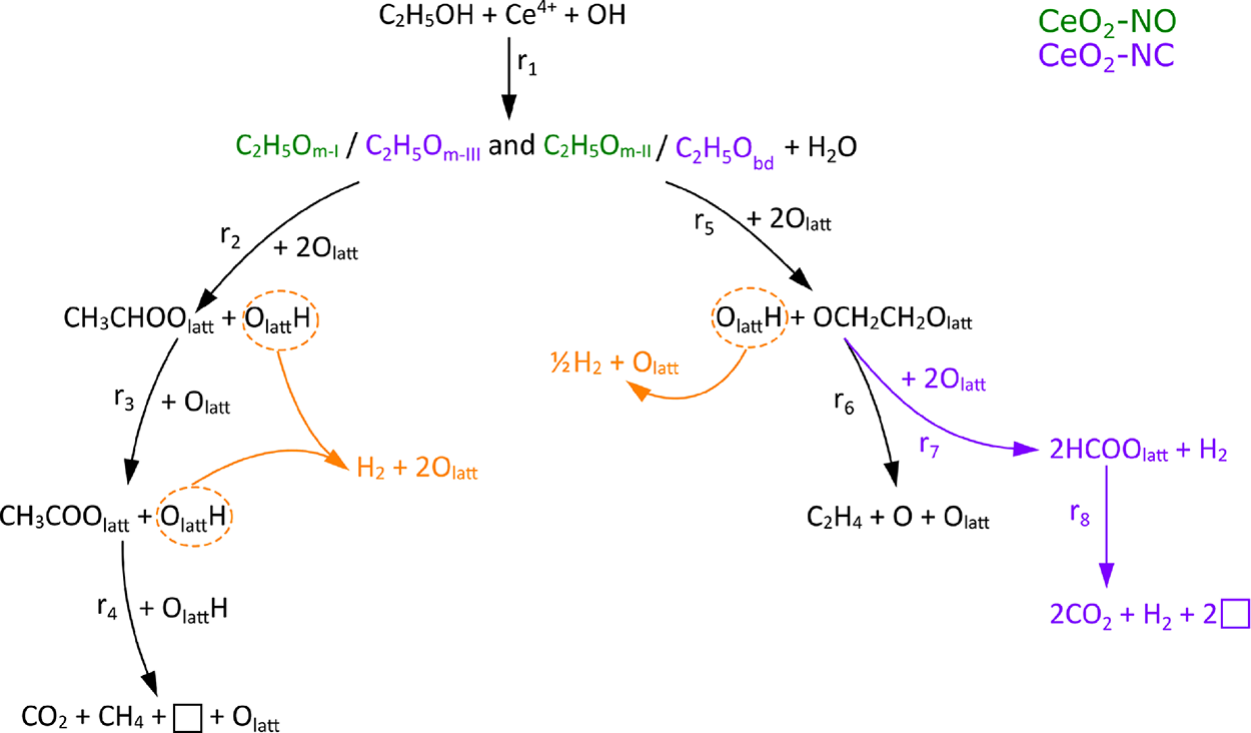
Mechanism for Ethanol Adsorption and Decomposition on CeO_2_-NO and CeO_2_-NC Ethoxy species in color green and purple correspond to CeO_2_-NO (C_2_H_5_O_m-I_ and C_2_H_5_O_m-II_) and CeO_2_-NC (C_2_H_5_O_m-III_ and C_2_H_5_O_m-bd_), respectively. Pathways in black and orange are common to both samples, while pathways in purple correspond only to CeO_2_-NC.

After ethanol adsorption at 100 °C, a number of bands assigned to ethoxy species can be detected on the surface of the CeO_2_ nanoshapes. The dissociative chemisorption of ethanol followed by water release is generally accepted as the first step of the mechanism for linear alcohol chemisorption on oxides (see Scheme 1, r_1_ in Section 3.2.3).^64,65^ The consumption of OH groups present at the surface can be observed by the negative bands in the 3800–3500 cm^–1^ region of the spectra after ethanol adsorption at 100 °C (Figure S2), in line with the reaction between the OH and H species to form water. The detailed assignment of each IR band is reported in Table 2 for the samples studied in this work. Figure 4 shows the IR spectra in the 1200–800 cm^–1^ region. The bands in this spectral region usually provide information on the coordination of alkoxy species on the surface of oxides.^66,67^ A clear difference can be found in the IR fingerprint of ethoxy species between CeO_2_-NO and CeO_2_-NC.

**Table 2:** Experimental and Calculated Infrared Frequencies (cm^–1^) Together with Surface Species and Mode Assignments during the Adsorption and TPSR of Ethanol on CeO_2_-NO and CeO_2_-NC

		experimental		calculated			
chemisorbed species	vibrational mode	CeO_2_-NO	CeO_2_-NC	C_2_H_5_O_m-I_^a^ (111)	C_2_H_5_O_m-II_^a^ (111)	C_2_H_5_O_m-III_ (100)-Mix	C_2_H_5_O_bd_ (100)-O
ethoxy	ν_as_(CH_3_)	2962	2965				
(C_2_H_5_O)	ν_as_(CH_2_)	2927	2930				
	ν_s_(CH_3_)	2864	2870				
	ν_s_(CH_2_)	2849	2850				
	δ(CH_2_) + Fermi resonance	2690	2704				
	δ(CH_2_)	n/o	1487				
	δ_as_(CH_3_)	1448	n/o				
	δ_s_(CH_3_)	1382	1384				
	ω(CH_2_)	1354	1355				
	ν(CCO)	1150	1152	1146	1135	1138	1148
	ν(CO)	1120	1118	1112		1106	
		1100	1096		1102		1096
	ν_as_(CCO)	1062	1061	1068		1071	
		1052	1045		1063		1041
	ν_s_(CCO)	905	905	904		915	
		889	882		898		883
acetate	ν_as_(OCO)	1552	1554				
(C_2_H_3_OO_latt_)	ν_s_(OCO)	1425	1428				
ethylenedioxy intermediate (OCH_2_CH_2_O_latt_)	ν(CH)	2833	2840		2831		2846
	ν(CO)	1041	(masked)		1039		1037
	ν(CC)	882	(masked)		881		874
Ce^3+^	forbidden electronic transition, ^2^F_5/2 →_^2^F_7/2_	2112	2110				

a The difference in the values of the frequencies with those reported in ref (48) is due to the fact that a different method was used to describe the long-range dispersion corrections.; n/o: not observed.

**Figure 5:**
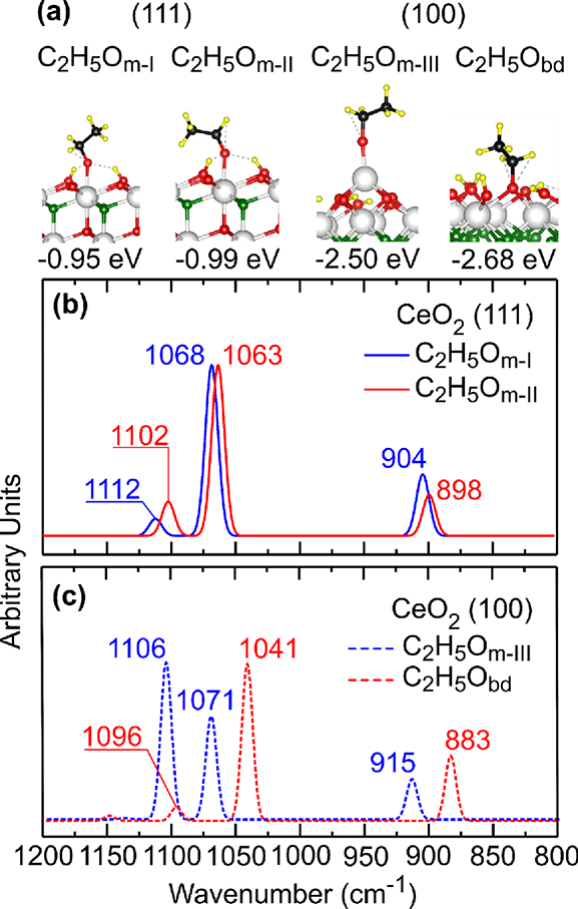
(a) Structures of ethoxy + H adsorbed on the hydroxylated ceria surface, where the adsorption energy is indicated with respect to the hydroxylated surface and gas-phase ethanol. (b,c) Simulated normalized IR spectra in the range of 1200–800 cm^–1^ for ethoxy + H species on the hydroxylated (111) and differently terminated (100) CeO_2_ surfaces, respectively.

**Figure 6:**
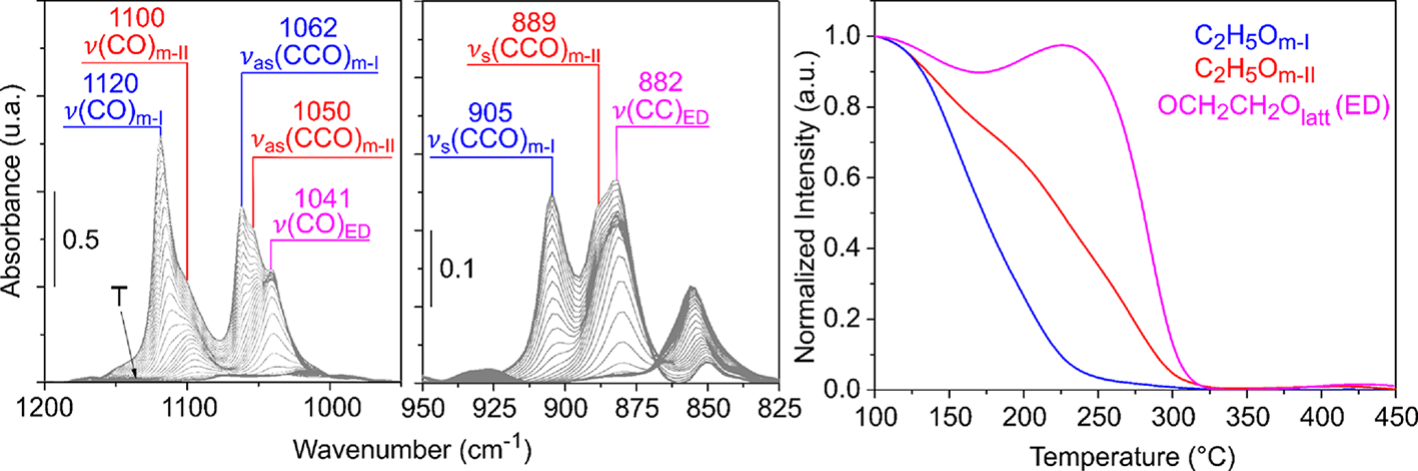
IR spectra and normalized integrated intensity of IR bands in the 1200**–**800 cm^–1^ region of the spectra during the TPSR-IR of ethanol for CeO_2_-NO.

In the case of CeO_2_-NO, a number of bands were identified at 1120, 1100, 1062, 1052, 1041, 905, 889, and 882 cm^–1^. The thermal evolution of these bands during the TPSR-IR experiments clearly showed that, on one side, the intensities of the signals at 1120, 1062, and 905 cm^–1^ decrease faster than the signals at 1100, 1052, and 889 cm^–1^, suggesting the presence of two types of ethoxy species with different thermal stability (see Section 3.2.3.1). As reported by some of us,^48^ DFT calculations on the hydroxylated (111) ceria surface, with one dissociated water molecule, allowed us to assign the first set of signals to monodentate ethoxy species in a standing-up (SU) configuration (with the C–C axis perpendicular to the surface) and the second set to monodentate ethoxy species in a lying-down (LD) configuration (with the C–C axis parallel to the surface), the former being the less stable one. For the sake of clarity, in this work, the SU and LD ethoxy species will be referred to as monodentate type I (C_2_H_5_O_m-I_) and type II (C_2_H_5_O_m-II_) ethoxy, respectively (see Figure 5a,b). The calculations in ref (48) were repeated with a slightly different computational setup (see Section 2.4). The newly optimized C_2_H_5_O_m-I_ and C_2_H_5_O_m-II_ structures are shown in Figure 5, and the corresponding vibrational frequencies are listed in Table 2. Based on the relative stability of these species (Figure 6), combined with the agreement between the simulated vibrational IR spectra in the range of 1200 to 800 cm^–1^ (Table 2), the assignment of C_2_H_5_O_m-I_ (less stable) and C_2_H_5_O_m-II_ (more stable) species to the faster and slower decomposing species, respectively, was made. On the other hand, the intensity of the signals at 1041 and 882 cm^–1^ have almost the same thermal evolution, decreasing more slowly than the ones of type I and type II monodentate ethoxy species. The assignment of those two bands will be addressed in the next section.

In the case of CeO_2_-NC, two types of ethoxy species can also be observed. A more labile species at 1118, 1061, and 905 cm^–1^ and a more stable species at 1096, 1045, and 882 cm^–1^, i.e., the latter set of frequencies, are red-shifted by up to 23 cm^–1^ compared to the former one (see Section 3.2.3.2). Moreover, comparing with the frequencies observed in the case of the nanooctahedra, it can be noted that the positions of the first set of IR signals in the nanocubes are almost identical to the ones of the more labile monodentate type I ethoxy species (C_2_H_5_O_m-I_) detected on the nanooctahedra (Figure 4). However, the frequencies within the second set of signals for the nanocubes are red shifted by up to 7 cm^–1^ compared to the analogous ones for the more stable monodentate type II species (C_2_H_5_O_m-II_) on the nanooctahedra.

DFT calculations of the stability and vibrational frequencies of ethoxy species were initially performed on the non-hydroxylated (100)-O ceria surface in order to identify the types of ethoxy species present on the CeO_2_-NC samples. Similar to the case of the (111) surface,^48^ we found that the calculated frequencies for the two more stable states on the (100)-O surface (Figure S3), which are of the bidentate type, do not reproduce the experimental findings for the CeO_2_-NC samples, namely, the three frequencies within the 1200–800 cm^–1^ range associated to the more stable species are not all red-shifted compared to those of the less stable species (cf. Table S2). In any case, we note that on the (100)-O surface, the oxygen of the ethoxy species can occupy the position of the lattice oxygen that has been removed from the surface layer, yielding a particularly stable bidentate surface species as compared to the monodentate ones on the (111) surface (Figure S3), in line with the findings by Beste and Overbury.^68^

Moreover, hydroxylating the (100)-O surface, as previously done for the (111) termination,^48^ adding in this case up to three dissociated water molecules (Figure S3), did not yield the expected result (cf. Table S2). With one water molecule, the question remains related to the relative shifts of the frequencies associated with the two more stable species found, i.e., no systematic red shift was obtained. With three water molecules (Figure S3), the set of frequencies corresponding to the most stable species are not red-shifted but blue-shifted, by up to 6 cm^–1^, compared to the set corresponding to the less stable one (Table S2). The most stable species on the hydroxylated (100)-O surface with three dissociated water molecules is of the bidentate standing up type with an adsorption energy of −2.68 eV, hereinafter referred to as C_2_H_5_O_bd_ (Figure 5).

To find another type of ethoxy species on the hydroxylated (100) surface, three dissociated water molecules were also added to the (100)-Mix facet (75%–25% mixture of the (100)-O and (100)-Ce terminations with c(2 × 2) periodicity), and the stability of differently coordinated and oriented ethoxy species was investigated. We recall that, as mentioned above, the stability of the (100)-Mix termination is comparable to that of the pure (100)-O. On this hydroxylated (100)-Mix termination, the most stable ethoxy species on the outermost Ce atom is of monodentate lying-down type (monodentate type III species, C_2_H_5_O_m-III_) with an adsorption energy of −2.50 eV (see Figure 5), that is, 0.18 eV less stable than the C_2_H_5_O_bd_ species already discussed. The calculated three frequencies within the 1200–800 cm^–1^ range for the less strongly bound C_2_H_5_O_m-III_ species, i.e., 1106, 1071, and 915 cm^–1^, are higher by up to 32 cm^–1^ compared to the corresponding ones for the more strongly bound C_2_H_5_O_bd_ species, i.e., 1096, 1041, and 883 cm^–1^ (cf. Figure 5c and Table 2). The relative stability of these two species and the magnitude of the relative shifts between the corresponding CO, asymmetric and symmetric CCO stretching vibrations, suggest that the experimentally observed more and less stable ethoxy species can be assigned to the C_2_H_5_O_bd_ and C_2_H_5_O_m-III_ species, respectively.

#### 3.2.3 Temperature Programed Surface Reaction by IR Spectroscopy (TPSR-IR)

After the adsorption of ethanol, the samples were heated under He flow to study the thermal evolution of the species adsorbed on the surface (TPSR-IR). The correlation of the TPSR results by IR in transmittance mode and by MS (see Section 3.2.1) together with DFT calculations allowed us to propose a mechanism for the reaction of ethanol with the surfaces of ceria studied in this work (Scheme 1), as discussed below. We first discuss the case of the CeO_2_ nanooctahedra and then that of the cubes in comparison to the former.

##### 3.2.3.1 CeO_2_-NO

Figure 6 shows the IR spectra during the TPSR-IR in the 1200–800 cm^–1^ region of the spectra (left and middle panel) and the thermal evolution (right panel) of the main bands (more intense and less overlapped) of ethoxy species on the CeO_2_-NO sample. As mentioned above, it can be clearly observed that the monodentate type I (C_2_H_5_O_m-I_) ethoxy is the more labile surface species that decomposes faster than the (more stable) monodentate type II (C_2_H_5_O_m-II_) ethoxy (*T*_50_ = 170 °C and *T*_50_ = 225 °C, respectively).

The 1041 and 882 cm^–1^ IR bands show a very unique behavior. While heating the sample from 100 to 175 °C, the intensity of these bands slightly decreases (10% decrease of the intensity). Then, above 175 °C, the intensity increases again until 230 °C, and finally sharply decreases, disappearing completely at 320 °C (see Figure 6 for the example of the 1041 cm^–1^ band). To understand this behavior and find the nature of the surface species associated with these bands, it is necessary to explore the IR spectra in the higher wavenumber region, that is, the one corresponding to the CH stretching vibrations [ν(CH)].

**Figure 7:**
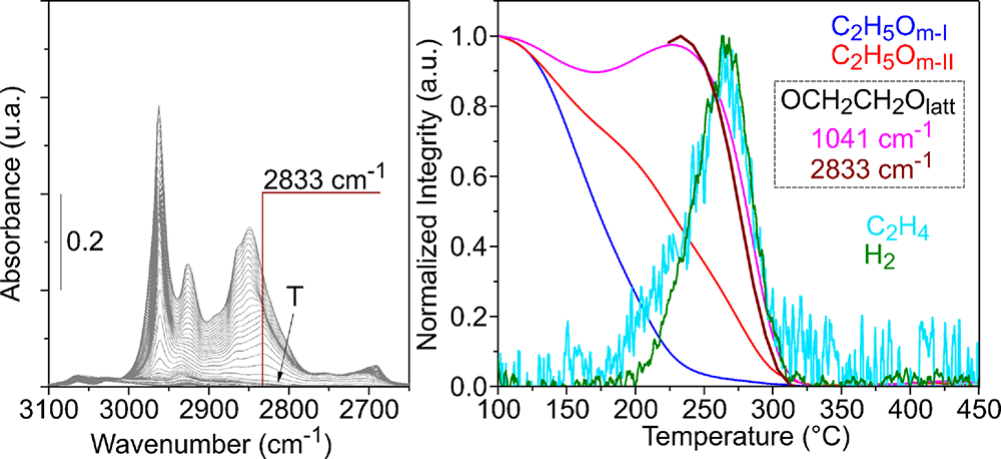
IR spectra in the 3100**–**2650 cm^–1^ region and normalized integrated intensity of the most important IR signals detected during the TPSR-IR and normalized intensity of the gaseous products detected during the TPSR-MS for CeO_2_-NO.

The left panel of Figure 7 shows the ν(CH) region of the spectra during TPSR-IR. In addition to the bands assigned to the ethoxy species (see Table 2), a signal at 2833 cm^–1^ begins to be detected above 175 °C. On the right panel of Figure 7, the thermal evolution of the normalized integrated intensity of this band is plotted together with that of the main bands in the 1200–800 cm^–1^ region and the evolution of gaseous ethylene and H_2_ (from the TPSR-MS experiments). Due to the band overlapping in the ν(CH) region of the spectra, the evolution of the peak at 2833 cm^–1^ cannot be clearly discriminated below 225 °C and, for this reason, the intensity of this band is only plotted from that temperature onward. However, above 225 °C, it can be observed that the intensity of the 2833 cm^–1^ IR band decreases at the same rate of the 1041 cm^–1^ signal, meaning that they might correlate to the same surface species.

In order to gain more information of the nature of the 2833 cm^–1^ IR band, an additional TPSR-IR experiment was carried out using ethanol with the deuterated methylene group, that is, CH_3_CD_2_OH (Figure S4). First, it can be noticed that a small band at 2830 cm^–1^ can be detected in the ν(CH) region of the spectra, without overlapping of other IR bands. In addition, a new band is detected in the ν(CD) region of the spectra at 2133 cm^–1^. The wavenumber ratio between 2830 and 2133 cm^–1^ is 1.32, which is very close to the one expected due to the isotopic exchange from C–H to C–D (this value is proportional to the square root of the reduced mass ratio of C–D to C–H, that is, 1.36). On the other hand, in the 1200–800 cm^–1^ region of the spectra (not shown), a peak at 970 cm^–1^ is detected, which shows a similar behavior to the 1041 cm^–1^ IR band detected during the TPSR of CH_3_CH_2_OH. Note that such a last relatively small isotopic shift correlates well with theoretical analysis, as exposed below. The normalized integrated intensities of the three IR bands, that is, 2830, 2133, and 970 cm^–1^, during the TPSR-IR of CH_3_CD_2_OH are plotted in Figure S4. It is clear that the thermal evolution of these three bands is almost identical, suggesting that they come from the same species, which most likely is a deuterated ethylenedioxy intermediate, OCH_2_CD_2_O_latt_, since the observed CH_2_ and CD_2_ vibrations (2830 and 2133 cm^–1^, respectively) and the CO vibration (970 cm^–1^) would correspond to such species. Moreover, we note that the maxima of the thermal evolutions of the 2833 and the 1041 cm^–1^ bands agree with the ∼50% decomposition of C_2_H_5_O_m-II_ species (Figure 7). In addition, when the intensity of these bands begins to decrease, ethylene is detected in the gas phase at the outlet of the reactor. In fact, the maximum of ethylene desorption matches with ∼40% conversion of the 2833 and 1041 cm^–1^ signals. Thus, the 2833 and 1041 cm^–1^ IR bands detected during the TPSR-IR of ethanol are assigned to an intermediate of ethylene production, referred to as ethylenedioxy intermediate. Therefore, it is proposed that the monodentate type II ethoxy (C_2_H_5_O_m-II_, the most stable one) species on the (111) surface of the CeO_2_-NO sample decomposes to form an ethylenedioxy intermediate (Scheme 1, r_5_) that later desorbs as ethylene in the gas phase (Scheme 1, r_6_), as suggested by the consecutive thermal evolutions of their IR representative bands at 1100 and 2833 cm^–1^ and of the ethylene MS signal (Figure 7). Another pathway proposed for ethylene production is by H addition to the α-C together with C–O bond cleavage of an enolate intermediate (CH_2_CHO–).^22^ However, evidence of an enolate intermediate is characterized by the C=C stretching peak at ∼1615 cm^–1^ while no such peak is detected in our spectra.

**Figure 8:**
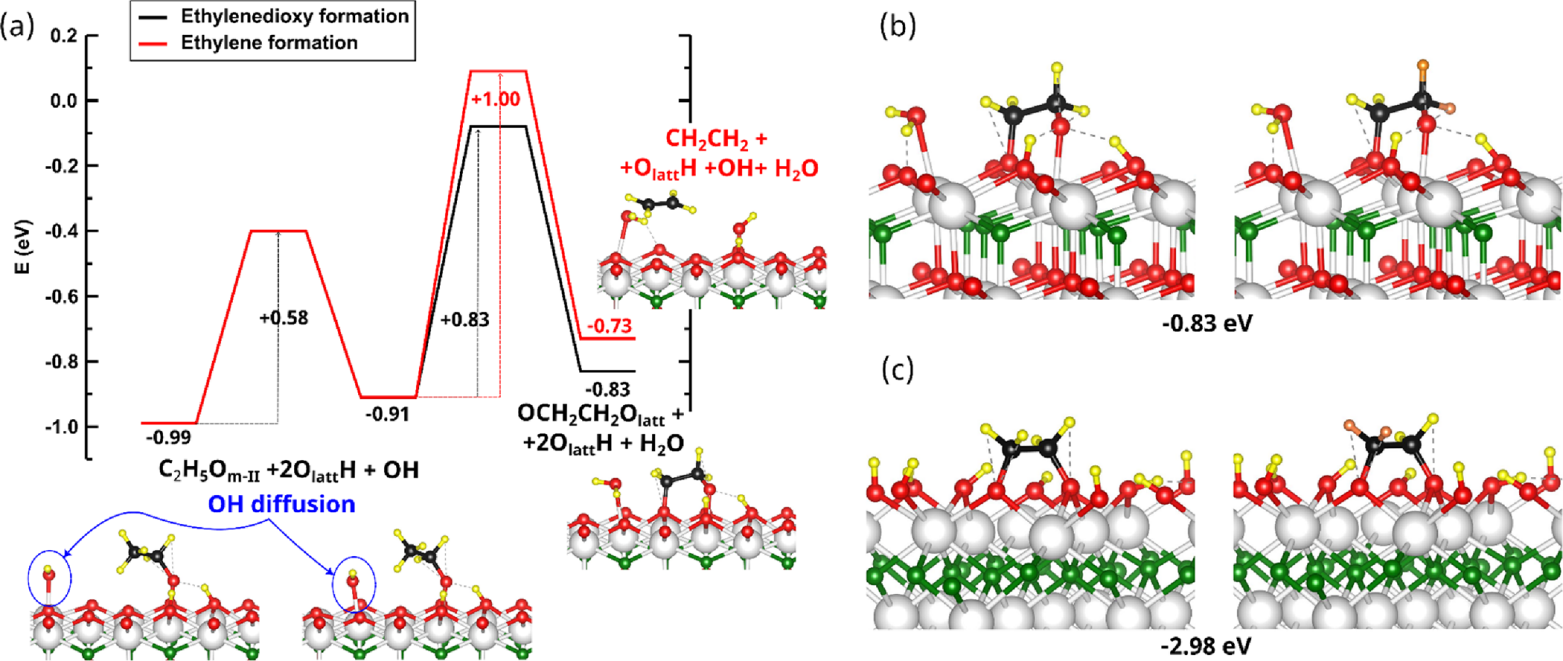
(a) Energy paths connecting the C_2_H_5_O_m-II_ state with the OCH_2_CH_2_O_latt_ intermediate and with the formation of adsorbed CH_2_CH_2_ for the example of the hydroxylated CeO_2_(111) surface. OCH_2_CH_2_O_latt_/OCH_2_CD_2_O_latt_ adsorbed on the hydroxylated (b) CeO_2_(111) and (c) CeO_2_(100)-O surfaces. The adsorption energies are referred to the ethanol/deuterated ethanol molecule in the gas phase and the hydroxylated surfaces, as explained in the Methods Section.

In order to get more insights into this ethylene intermediate, DFT calculations were performed for an ethylenedioxy species (hereinafter referred to as OCH_2_CH_2_O_latt_). For the case of the hydroxylated CeO_2_(111) surface, we have searched for the minimum energy path connecting the C_2_H_5_O_m-II_ state with the OCH_2_CH_2_O_latt_ intermediate and compared it with that for the formation of adsorbed CH_2_CH_2_ (Figure 8a). Both paths have two steps and involve the same first step where a OH species diffuses between neighboring Ce atoms. In the second step, the formation of the ethylenedioxy intermediate involves the simultaneous β-H abstraction from the C_2_H_5_O_m-II_ species, which will form a H_2_O molecule with the OH that has diffused, and the binding of the β-C with an O_latt_. The formation of adsorbed ethylene also occurs via a β-H abstraction pathway that forms a H_2_O molecule, which is accompanied by the cleavage of an O_latt_–H bond. The cleaved H binds to the O of the ethoxy, causing the cleavage of the O–C bond and the formation of ethylene and a OH species. The OH diffusion has a low activation barrier of 0.58 eV, and therefore diffusion of hydroxyl species is feasible under experimental reaction conditions. Second, the ethylenedioxy formation has an activation barrier that is 17% lower than that corresponding to ethylene formation. These results are further evidence, together with the TPSR-IR and DFT results of OCH_2_CH_2_O_latt_/OCH_2_CD_2_O_latt_, that these species are intermediates in the decomposition of ethanol.

Frequencies for the OCH_2_CH_2_O_latt_ (Figure 8b) on the hydroxylated CeO_2_(111) surface were calculated, finding values of 2831, 1094, 1039, and 891 cm^–1^ (Figure S5a). The calculated frequencies of 2831 and 1039 are in good agreement with the reported experimental values of 2833 and 1041 cm^–1^, corresponding to ν(CH) and ν(CO) stretching vibrations, respectively, whereas the frequencies of 1094 and 891 cm^–1^ lie within the 1112–1102 and 904–898 cm^–1^ ranges, within which frequencies of ethoxy species have been found. Moreover, the spectrum of the deuterated ethylene species (Figure 8b) was also simulated with the three most intense frequencies being 2821, 2133, and 985 cm^–1^ (see Figure S5a). By comparing to the non-deuterated species, we observe that the 2831 cm^–1^ band in the ν(CH) region of the spectra appears at 2133 cm^–1^ in the ν(CD) region (ratio 1.32), in line with the experimental results and thus unambiguously establishing the presence of an ethylenedioxy intermediate.

A point that probably deserves to be highlighted is the detection of the ethylenedioxy intermediate from the beginning of the TPSR-IR experiments. Either deuterated or not deuterated ethanol seems to decompose to give the ethylenedioxy intermediate on the surface of the ceria nanooctahedra, even at 100 °C (see Figure 7 and Figures S4). As the temperature begins to rise, a small fraction of that intermediate desorbs to give ethylene. Simultaneously, the ethylenedioxy intermediate is produced from the decomposition of C_2_H_5_O_m-II_ at temperatures higher than 175 °C, giving rise to the valley observed at 175 °C (Figure 7). In fact, as mentioned in Section 3.2.1, a low temperature shoulder of gaseous ethylene begins to develop above 175 °C during the TPSR–MS, which could be related to the transformation of the ethylenedioxy intermediate below 175 °C. Li et al. observed a broad desorption peak of ethylene at around 130 °C on ceria nanooctahedra after ethanol TPD experiments and suggested that a low-energy concerted bimolecular elimination pathway (E2) can occur from adsorbed ethanol.^22^ However, our explanation seems to be more consistent due to the direct experimental IR evidence of the proposed ethylenedioxy intermediate from 100 °C.

**Figure 9:**
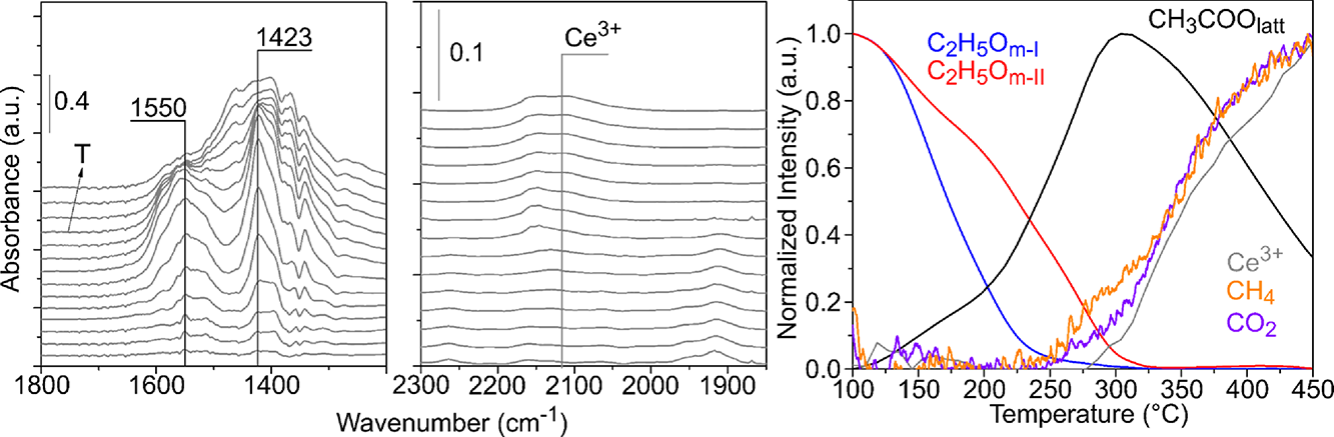
IR spectra in the 1800**–**1200 and 2300–1850 cm^–1^ regions and normalized integrated intensity of the most important IR signals detected during the TPSR-IR and normalized intensity of the gaseous products detected during the TPSR-MS for CeO_2_-NO.

In the 1800–1200 cm^–1^ region of the spectra, two signals at 1550 and 1423 cm^–1^, assigned to acetate species (CH_3_COO_latt_), can be detected above 125 °C (Figure 9, left panel). The thermal evolution shows that the intensity of these signals increases until 300 °C (Figure 9, right panel). Next, acetate species begin to decompose while at the same time, CO_2_ and CH_4_ are observed at the outlet of the reactor. Additionally, the signal at 2130 cm^–1^, assigned to the forbidden electronic transition ^2^F_5/2_ → ^2^F_7/2_ of Ce^3+^,^69^ shows a similar development as CO_2_ and CH_4_. In the case of polycrystalline ceria,^48^ it was proposed that acetate may be formed by two simultaneous α-CH scissions of C_2_H_5_O_m-I_ species (Scheme 1, r_2_ and r_3_), that is, of the more labile surface ethoxy, since the formation of acetate species matched 50% of the conversion of the type I ethoxy species. However, acetate formation on the CeO_2_-NO surface does not intersect at 50% of the C_2_H_5_O_m-I_ depletion (Figure 9). This discrepancy can be explained by assuming that in our model nanooctahedra material, two consecutive α-CH scissions are responsible for the acetate formation while on a defected powder surface (like polycrystalline ceria), both elementary reaction steps could not be discerned. Above 300 °C, acetate species begin to decompose releasing CO_2_ and CH_4_ in the gas phase leaving an oxygen vacancy at the surface (Scheme 1, r_4_).

Finally, the gaseous hydrogen detected during the TPSR-MS (Figure 3) could be the result of the recombination of H and/or OH species resulting from both the dehydrogenation of C_2_H_5_O_m-II_ ethoxy species to the ethylenedioxy intermediate (Scheme 1, r_5_) and the formation of acetate species from C_2_H_5_O_m-I_ (Scheme 1, r_2_ and r_3_).

##### 3.2.3.2 CeO_2_-NC

**Figure 10:**
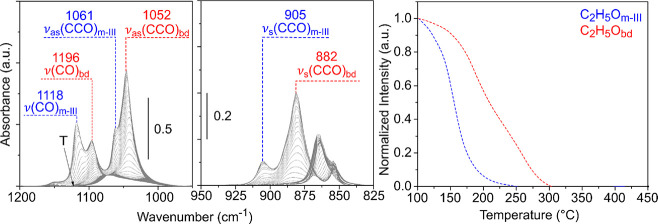
IR spectra and normalized integrated intensity of IR bands in the 1200**–**800 cm^–1^ region of the spectra during the TPSR-IR of ethanol for CeO_2_-NC.

Figure 10 shows the thermal evolution of the ethoxy species adsorbed on CeO_2_-NC. As discussed in Section 2.2, on the nanocubes, two species have been identified, namely, C_2_H_5_O_m-III_ and C_2_H_5_O_bd_. It can be observed that the monodentate C_2_H_5_O_m-III_ species decomposes faster (*T*_50_ = 155 °C) than the bidentate C_2_H_5_O_bd_ species (*T*_50_ = 207 °C).

**Figure 11:**
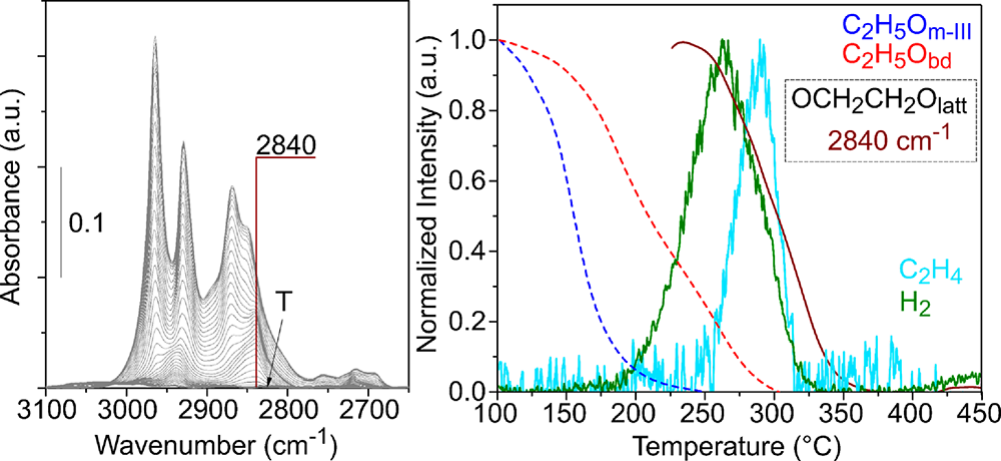
IR spectra and normalized integrated intensity of IR bands in the 3100**–**2650 cm^–1^ region of the spectra during the TPSR-IR of ethanol and normalized intensity of the gaseous products detected during the TPSR-MS for CeO_2_-NC.

A chemisorbed OCH_2_CH_2_O_latt_ species was also detected on the CeO_2_-NC. Importantly, we found that the OCH_2_CH_2_O_latt_ species are about 2.15 eV more stable on the (100) surface (Figure 8). An experimental (theoretical) signal at 2840 (2846) cm^–1^ was recorded in the ν(CH) region of the spectra (Figure 11 and Figure S5). However, a signal at approximately 1040 (1037) cm^–1^ could not be discerned because it is probably completely masked by the presence of IR signals assigned to bidentate ethoxy species. A TPSR-IR experiment after adsorption of deuterated CH_3_CD_2_OH molecules was also performed (Figure S6). Two signals at 2837 and 2140 cm^–1^ (calculated at 2847 and 2151 cm^–1^, respectively, Figure S5b) in the C–H(D) stretching region can be clearly observed and show similar evolutions, attributed to the ν(CH) and ν(CD) modes, respectively, of the chemisorbed OCH_2_CD_2_O_latt_ species.

Now, going back to the evolution of the 2840 cm^–1^ IR band of the non-deuterated ethylenedioxy species during the TPSR-IR of ethanol plotted in Figure 11, it can be observed that this signal reaches a maximum at 230 °C, which agrees with a 60% conversion of the C_2_H_5_O_bd_ ethoxy species. Thus, in the case of CeO_2_-NC, it is proposed that bidentate ethoxy species are the ones that react to form chemisorbed OCH_2_CH_2_O_latt_ species (Scheme 1, r_5_), which later on partially release ethylene to the gas phase (Scheme 1, r_6_). It is worth mentioning that the OCH_2_CH_2_O_latt_ species decompose at higher temperature on CeO_2_-NC than on CeO_2_-NO (cf. Figure 7, *T*_50_ is 300 and 280 °C for the decomposition on nanocubes and nanooctahedra, respectively) and, as a consequence, ethylene is also released at higher temperatures on the nanocubes, reaching a maximum concentration at 295 °C (Figure 11) and consistent with TPSR-MS results (Figure 3).

As mentioned in Section 3.2.1, the amount of ethylene released in the gas phase is 1.75-times (Figure 3) larger on CeO_2_-NO than on the CeO_2_-NC. To correlate the ethylene production with the concentration of the OCH_2_CH_2_O_latt_ species, the maximum integrated intensity of the ν(CH) and ν(CD) IR bands of the OCH_2_CD_2_O_latt_ species was normalized by the surface area of each ceria wafer. The intensity ratio of each IR stretching mode showed that the ethylenedioxy surface concentration is two times smaller on the ceria nanooctahedra than on the nanocubes for both the CH and the CD vibrations, which is opposite to the corresponding ethylene concentrations in the gas phase. The fact that the ethylenedioxy concentration is smaller on the CeO_2_-NO while the ethylene released in the gas phase is larger as compared to the CeO_2_-NC can be related to the lower binding (by 2.15 eV, Figure 8) of that intermediate on the (111) facets, facilitating the desorption of ethylene to the gas phase.

**Figure 12:**
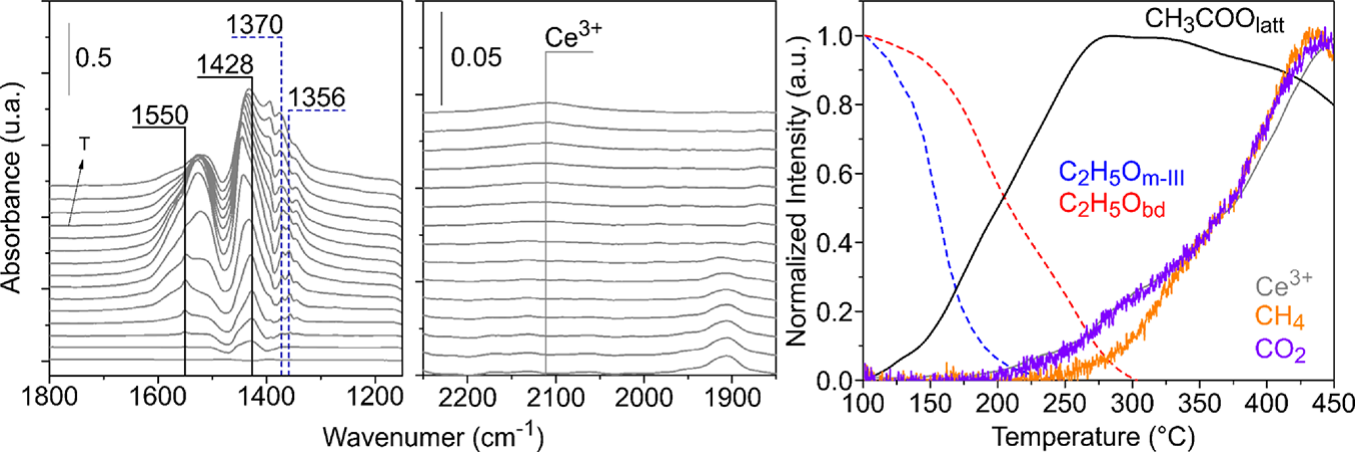
IR spectra in the 1800–1200 and 2300–1850 cm^–1^ regions and normalized integrated intensity of the most important IR signals detected during the TPSR-IR and normalized intensity of the gaseous products detected during the TPSR-MS for CeO_2_-NC.

Furthermore, the presence of two additional peaks at 1370 and 1356 cm^–1^ is detected for the CeO_2_-NC sample (see Figure 12), which are attributed to formate surface species. It is proposed then that some of the particularly stable OCH_2_CH_2_O_latt_ species on the (100) facets react on the surface of ceria nanocubes, by a parallel pathway, to give formate species (pathways in purple, Scheme 1, r_7_). Those surface formate groups decompose to CO_2_ and H_2_, leaving two oxygen vacancies on the surface (pathways in purple, Scheme 1, r_8_), which is correlated to the CO_2_ shoulder at 280 °C and the evolution of Ce^3+^ in Figure 12. Another experimental result agrees with this last proposal: formate species are not detected in the CeO_2_-NO sample for which the ethylenedioxy intermediate is less stable. Moreover, the oxidation of the OCH_2_CH_2_O_latt_ species to formate species is in line with the greater ease with which oxygen vacancies are formed on the CeO_2_(100) surface compared to the (111).^49^ That is, the ethylenedioxy intermediate on (100) facets of the nanocubes seems to be more sensitive to be attacked (oxidized) by surface oxygen than the one on the (111) facets of the nanooctahedra.

Acetate species were also observed on the surface of the nanocubes. The signals at 1550 and 1428 cm^–1^ are detected from 110 °C, both reaching their maximum at approximately 280 °C (Figure 12). Since the corresponding signals for acetate species on the nanooctahedra are detected above 125 °C and their intensity increases until 300 °C (cf. Figure 9), it can be concluded that the formation and decomposition of acetate species occur faster on the CeO_2_-NC than on the CeO_2_-NO. Then, it is proposed that acetate species are the product of two α-CH scissions of the more labile monodentate C_2_H_5_O_m-III_ ethoxy species (Scheme 1, r_2_ and r_3_). As in the case of ceria nanooctahedra, it is suggested that a CH_3_CHOO_latt_ intermediate may be formed after the first α-CH scission that is not detected in the IR spectra (Scheme 1, r_2_) and for this reason, the evolution of the IR signals of C_2_H_5_O_m-III_ and acetate species shown in Figure 12 does not match 50% of the conversion as expected. Again, Ce^3+^, CO_2_, and CH_4_ are produced as soon as acetate species begin to decompose (Scheme 1, r4). Figure S7 shows the evolution of acetate species on CeO_2_-NO and CeO_2_-NC during the TPSR-IR, normalized by the surface area of each wafer. It can be observed that the amount of surface acetate species is higher on nanocubes than on nanooctahedra. This is consistent with the higher release of gaseous CO_2_ and CH_4_ on the cubes. This, together with the formation of formate species on the cubes, indicates that on the surface of the CeO_2_-NC, the C–C bond can be more easily cleaved than on the CeO_2_-NO. In other words, CO_2_ production on CeO_2_-NC can come from both C_2_H_5_O_m-III_ and C_2_H_5_O_bd_ ethoxy species, which further decompose to chemisorbed acetate and ethylenedioxy intermediate, respectively, followed by C–C cleavage in both cases. However, on CeO_2_-NO, only one reaction pathway is possible toward CO_2_ production, which involves C_2_H_5_O_m-I_ oxidation to acetate species (cf. Scheme 1).

We further note that, according to the mechanism for ethanol decomposition proposed in Scheme 1, CeO_2_-NC produces two additional oxygen vacancies on the surface compared to CeO_2_-NO (pathway in purple, Scheme 1, r_8_). In line with this, we show that the integrated intensity of the Ce^3+^ signal as a function of temperature, plotted in Figure S8, reflects that the concentration of Ce^3+^ is higher on the nanocubes compared to the nanooctahedra throughout the whole temperature range. In fact, at 450 °C, the integrated intensity of the Ce^3+^ signal is 2.2-fold larger on the cubes than on the octahedra. This is in agreement with the aforementioned greater ease with which oxygen vacancies form on the CeO_2_(100) surface compared to (111).^49^

The other product of ethanol reforming is H_2_. In the case of ethanol decomposition, the molecular hydrogen evolutions are almost the same in both nanoshapes, meaning that the recombination of H and/or OH species is similar on both types of surfaces (pathways in orange, Scheme 1). The higher H_2_ released in the gas phase in the case of CeO_2_-NC (see Section 3.2.1) can be explained by two additional sources of H_2_ on the cubes, that is, the decomposition of the ethylenedioxy intermediate into formate species (Scheme 1, r_7_), which are further decomposed (Scheme 1, r_8_).

### 3.3 Ethanol Steam Reforming over CeO_2_ Nanoshapes

**Figure 13:**
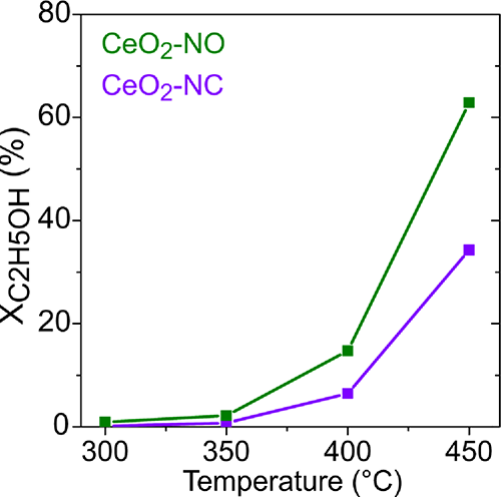
Ethanol conversion vs temperature (300–450 °C) for CeO_2_-NO and CeO_2_-NC (H_2_O:C_2_H_5_OH = 6:1, 800 m^2^ h/mol C_2_H_5_OH).

Figure 13 shows the ethanol conversion as a function of temperature for CeO_2_-NO and CeO_2_-NC. It can be observed that both ceria nanoshapes start to convert ethanol above 350 °C. Additionally, the ethanol conversion is higher on CeO_2_-NO than on CeO_2_-NC in the whole temperature range, reaching maximum conversions of 63 and 34%, respectively, at 450 °C. The H_2_ molar fraction and the product yields during the ESR experiment at 400 and 450 °C, that is, temperatures for which significant ethanol conversions (7–63%) were reached, are shown in Figure S9. The main byproducts under ethanol reforming conditions on both samples are ethylene, acetaldehyde, acetone, and CH_4_. However, the product distributions are different between both ceria nanoshapes. At 400 and 450 °C, ethylene yields were 4.1 and 2.5-times higher, respectively, on the CeO_2_-NO than on CeO_2_-NC. On the other hand, higher CO_2_ yields were achieved on the nanocubes compared to the nanooctahedra (20 and 40% higher at 400 and 450 °C, respectively), while the H_2_ molar fraction was almost identical on both samples. As a consequence, the H_2_:CO_2_ ratios at the outlet of the reactor were 8.6 and 4.4 for CeO_2_-NO and CeO_2_-NC, respectively, at 450 °C. Even though the ethanol conversions were different between both ceria nanoshapes, the H_2_:CO_2_ for the CeO_2_-NC sample is closer to the theoretical value expected for the ESR reaction (H_2_:CO_2_ = 3), and then the ceria nanocubes are expected to be better reforming catalyst than nanooctahedra. Thus, the higher conversions achieved for the CeO_2_-NO are due mainly to the formation of ethylene, that is, the dehydration of ethanol rather than its reforming. We note that Soykal and collaborators^24^ studied bare ceria nanorods and nanocubes under ESR conditions (H_2_O:EtOH = 10:1, WHSV = 2.03 g_C_2_H_5_OH_/(g_cat_ h), that is, *S*_CeO_2__/*F*°_C_2_H_5_OH_ = 1370 m^2^ h/mol_C_2_H_5_OH_) and found that the nanocubes were more active at all temperatures tested (350–500 °C). They also found that while acetaldehyde was the main product over the ceria nanorods, the nanocubes showed better C–C bond cleavage activity, producing higher H_2_ yields and higher CO_2_ selectivity at all temperatures tested.

**Figure 14:**
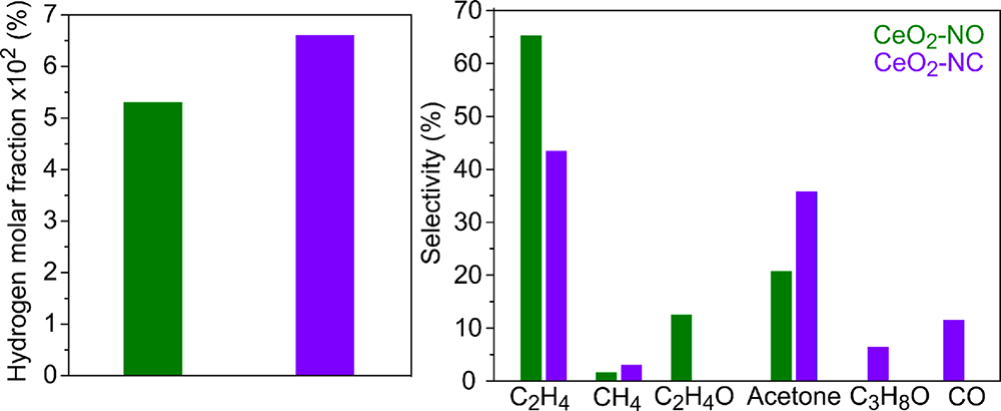
Ethanol reforming under differential conditions (400 °C). *X*_C_2_H_5_OH_: 5.4 and 4.0%, and *S*_CeO_2__/*F*°_C_2_H_5_OH_ = 530 and 210 m^2^ h/mol_C_2_H_5_OH_ for CeO_2_-NO and CeO_2_-NC, respectively, in each case.

In order to get a deeper insight into the reactivity differences between CeO_2_ nanocubes and nanooctahedra, an additional activity measurement was performed under differential conditions (approximately 5% ethanol conversion for the cubes and octahedra at 400 °C). The H_2_ molar fraction and the selectivities plotted in Figure 14 show that the octahedra are more selective toward ethylene and acetaldehyde, whereas the cubes produce more H_2_, CH_4_, CO, acetone, and isopropyl alcohol. These results are in agreement with the TPSR of ethanol, where CeO_2_-NO also showed a higher production of ethylene in the absence of water, while CeO_2_-NC showed a greater capability to break the C–C bond, producing higher amounts of CH_4_ and CO_2_. Strikingly, CO_2_ was not detected under differential ESR conditions, probably due to the adsorption of the small fractions of CO_2_ produced, which can be (partially) trapped as carbonate species.

The activation energies (*E*_a_) for ethylene and acetone production, that is, the main products of the ESR reaction on CeO_2_-NO and CeO_2_-NC, were measured in the range of 380–430 °C (see Figure S10). In the case of ethylene production, the activation energies are ∼180 kJ/mol for both ceria nanoshapes studied in this work. These results suggest that the rate-limiting step for the ethylene production might be the same on both ceria nanoshapes. Therefore, the higher production of ethylene by the ceria nanooctahedra (see Figure S10) could be due to a greater concentration of surface species responsible for the formation of ethylene. Although during the TPSR-IR, the concentration of ethylenedioxy intermediate was higher on the nanocubes than on the nanooctahedra, on the surface of the former, some of this species give rise to formate species that decompose into gas phase CO_2_ and H_2_.

The values of the activation energies for acetone production are almost identical on the CeO_2_ nanocubes as compared to the CeO_2_ nanooctahedra (139 and 153 kJ/mol, respectively). However, if we assume that acetate species are involved in acetone production under ESR by the ketonization reaction involving surface acetate species, which is the main hypothesis of several authors,^70^ it is possible to correlate the higher surface concentration of acetate observed by TPSR-IR on the nanocubes to the higher acetone selectivity under ESR conditions (cf. Figure S7 and Figure 14). To fully elucidate the ESR reaction mechanisms, further studies under operando conditions that also take into account the role of Ce^3+^, which can be present at *T* > 300 °C, and of water would be necessary. Either way, it is clear that ceria nanooctahedra show higher ethanol conversions than nanocubes during the ESR experiments.

Thus, both the TPSR and reactivity experiments indicate that the CeO_2_(111) surface is more active toward ethylene production, that is, ethanol dehydration. On the other hand, the nanocubes revealed lower ethanol conversion, though the CeO_2_(100) surface appears to be better than the (111) at breaking the C–C bond and is more selective toward H_2_ and CO_2_, in other words, superior for ethanol reforming. At this point, it is worth mentioning that surface reconstruction of nanoshaped ceria particles has been reported.^20,71−74^ In particular, nanofaceting is known to occur on the edges of ceria nanocubes after thermal treatment under hydrogen (*T* > 500 °C, 3 h) and under oxygen (*T* > 600 °C, 3 h), the nanooctahedra being very stable at those reducing and oxidizing atmospheres, respectively (up to 700 °C).^73^ However, since our pretreatment was performed at 450 °C (under H_2_, He, and O_2_, 15 min each), we do not expect to have major reconstruction on the edges of our CeO_2_-NC sample, which could account for 10% of the total exposed surface.^71^ In any case, the effect of surface restructuring of ceria nanoshapes is a matter of further systematic work. In summary, the use of ceria nanoshapes constitutes an adequate model to study the interaction of ethanol with different ceria surfaces with well-defined crystal planes. By a combination of experimental and theoretical results, together with the use of ceria with different morphologies, we have been able to establish a correlation between the surface structure and the ESR activity of the different ceria nanoshapes.

## 4 Discussion

In this section, our results are discussed in light of the results in the literature for model ESR ceria catalysts, namely, single crystals and ceria nanoshapes.^22,68,75−77^ As shown in Scheme 1, the surface structure of cerium oxide was found to influence ethanol reaction routes. Mainly, it was shown that during ethanol decomposition, ceria nanocubes, dominated by (100) surface crystal planes, are better at breaking the C–C bond of ethanol compared to the nanooctahedra, which mainly expose (111) planes. A correlation was found between those results and the catalytic performance toward ESR of the ceria nanoshapes. Namely, the C–C bond cleavage capacity of CeO_2_-NC improved the selectivity toward H_2_ and CO_2_ compared to CeO_2_-NO.

Ethanol adsorption and decomposition have mainly been investigated over ceria thin films under UHV conditions,^75,76^ while the use of bare ceria nanoshapes, that is, without the metallic function, is rather scarce. Li and coworkers^22^ have studied the ethanol adsorption over ceria nanorods, nanocubes, and nanooctahedra by means of DRIFT spectroscopy. Infrared bands attributed to surface ethoxy species were intense on the rods and nanocubes but rather weak on the nanooctahedra. In fact, after the evacuation of ethanol by flowing He through the DRIFT cell, the IR bands in the 1200–800 cm^–1^ region of the spectra drastically decreased in their CeO_2_ nanooctahedra. It must be taken into account that their nanocubes have 2-fold higher *S*_BET_ surface area compared to their nanooctahedra (27 vs 13 m^2^/g, respectively). However, normalizing the IR spectra by the surface area of their samples does not account for the differences in the intensity of the ethoxy signals. In the case of our ceria nanoshapes, if we integrate the IR spectra in the 1200–950 cm^–1^ region after ethanol adsorption and evacuation, normalized by the area of the sample loaded in our IR cell (see Figure 4), that IR area on the CeO_2_-NC is only 1.15-fold higher than on CeO_2_-NO. This discrepancy found between the behavior of the ceria nanooctahedra reported by Li et al.^22^ and the one presented in our work could be due to the different synthesis methods employed. As mentioned in the Experimental Section, the hydrothermal method employed here does not use phosphate, which is known to have an impact on the properties of CeO_2_.^38^ López-Granados et al. have proposed that two different domains exist when P is incorporated in CeO_2_, depending on the P/Ce ratio.^38^ For P/Ce values <0.03, isolated orthophosphate units are proposed on the ceria surface and within the subsurface layers of the phosphated Ce oxide, which are responsible for the inhibition of oxygen diffusion within the subsurface region. At higher P/Ce ratios, CePO_4_ (monazite) crystals are detected in the surface, that is, a very stable Ce^3+^ phase unable to participate in the Ce^4+^/Ce^3+^ redox couple required for the optimum functioning of the oxygen storage and release (OSR) properties of ceria. Either the formation of orthophosphate species and/or monazite crystals could be responsible for blocking adsorption sites for ethoxy species, which would in turn result in lower intensity of the observed DRIFT signals on the nanooctahedra reported by Li et al.^22^

The temperature-programmed decomposition of ethanol followed by mass spectroscopy was also studied by Li and coworkers over the three ceria nanoshapes.^22^ In the case of their ceria nanocubes, similar results were obtained compared to our CeO_2_-NC sample. However, some differences can be found in the case of the nanooctahedra. According to Li et al., two broad and unresolved ethylene desorption peaks between 70 and 330 °C were reported.^22^ Additionally, only a small amount of CH_4_ was observed at the outlet of the reactor, and H_2_ evolution showed a maximum at 380 °C, that is, 100 °C higher compared to our ceria nanooctahedra. As shown in our Scheme 1, the participation of ceria lattice oxygen is proposed to be crucial in every step of the ethanol reaction on the surface of our CeO_2_ nanoshapes. The formation of both acetate and ethylenedioxy species that later on produce CO_2_ and CH_4_ or ethylene, respectively, involves surface lattice oxygen. Thus, the modification of the OSR properties of ceria in the presence of P on the surface could be responsible for the differences in the ethanol decomposition between our results and the ones reported by Li and collaborators on ceria nanooctahedra.^22^

Another difference worth mentioning is that neither ethanol nor acetaldehyde was detected in the gas phase for any of our ceria nanoshapes. Conversely, Li et al. detected ethanol and acetaldehyde as broad features between 70 and 250 °C.^22^ These contrasting results could be explained by the different adsorption temperatures used in our experiments. As reported recently by some of us, a TPSR-MS experiment performed after ethanol adsorption at 25 °C over polycrystalline ceria showed broad desorption peaks of ethanol and acetaldehyde between room temperature and 150 °C.^48^ Thus, it is proposed that during ethanol adsorption at 100 °C followed by purging with He, most of the acetaldehyde has already been removed from our reactor, that is, before our study of ethanol decomposition, which is governed by ethoxy species.

Then, two types of ethoxy species with different thermal stability were identified by means of IR spectroscopy on each ceria nanoshape. Over the CeO_2_(111) surface, DFT calculations identified those ethoxy species as monodentate type I and II ethoxy with the alkyl chain more parallel or more perpendicular to the surface. On the CeO_2_(100) surface, bidentate and monodentate type III ethoxy species are proposed on the checkerboard O termination and on a pyramid of the reconstructed (100) surface, respectively. Then, we have proposed that the more stable surface ethoxy species on each ceria nanoshape, which are the monodentate type II and bidentate ethoxy, are responsible for the formation of an ethylenedioxy intermediate. An ethylenedioxy intermediate was also proposed over CeO_2_(111) thin films after the adsorption of ethylene glycol by means of the XPS C 1s spectrum.^76,77^ In contrast, Mullins’ and Libuda’s groups did not detect such an intermediate after ethanol adsorption and reaction over CeO_2_(111).^71,72^ Moreover, Lykhach et al. were not able to observe acetate species by means of XPS after ethanol adsorption and reaction on CeO_2_(111) thin films and suggested the presence of an acetaldehyde-like intermediate.^76^ However, using infrared spectroscopy, the surface acetate group was revealed over our CeO_2_ nanoshapes and we propose its formation from two consecutive α-CH scissions of monodentate type I or III ethoxy (on CeO_2_-NO and CeO_2_-NC, respectively). At this point, even though the origin of both discrepancies is not clear, our IR experimental evidence that agrees with the DFT results, including isotopic labeling, suggests the presence of ethylenedioxy species and acetate species during ethoxy decomposition.

Three considerations must be taken into account in order to understand the differences found in our study with ceria nanoshapes and the studies performed over CeO_2_(111) thin films. On the one hand, the experimental conditions are very different in both kinds of experiments. In the work of Mullins et al. and Lykhach et al., ethanol adsorption was performed at −88 and −123 °C, respectively,^75,76^ whereas in this work, it was at 100 °C. On the other hand, the experimental techniques used for the identification of adsorbed surface species are based on contrasting principles. IR spectroscopy as used in this work is a more suitable technique for identifying adsorbed surface species since the vibrations provide a direct fingerprint of the molecular fragments, whereas XPS as used by Mullins et al. and Lykhach et al.^75,76^ is an indirect technique that measures shift in the binding energy of the C 1s electrons, which is affected by the surrounding bonds of the C atom. Finally, the truncated vertices that expose (100) facets observed on our nanooctahedra, together with edges and corners sites, could also be contributing to the different experimental results compared to CeO_2_(111) model catalysts. The ethanol adsorption temperature could probably affect the nature of the released products at low temperature since the surface is covered by molecular species, some of them partially physisorbed, giving rise to ethanol and acetaldehyde as explained before. The experimental technique and the existence of the truncated vertices on our nanooctahedra could be playing a role in the interpretation of the results and the results themselves, respectively. However, the IR intensity of the proposed intermediates (ethylenedioxy and acetate) is large enough as to have originated from a small proportion of defects on the nanoshapes.

Furthermore, the pathways for ethanol dehydrogenation and dehydration catalyzed by ceria extended non-hydroxylated (111) and (100) surfaces have been studied by computational methods by Beste and Overbury.^68^ For both surfaces, they identified a one-step pathway for ethylene formation, where β-H transfer occurs simultaneously with O–C bond scission as well as a two-step pathway, where β-H transfer occurs first leading to an intermediate (•CH_2_CH_2_O) that further reacts through O–C scission. On the other hand, they proposed that acetaldehyde formation can involve α-H–C scission or an intramolecular transfer of a hydrogen atom of the •CH_2_CH_2_O intermediate. They proposed that acetaldehyde formation through α-H – C scission is on both surfaces the energetically lowest reaction pathway. The selectivity for acetaldehyde and ethylene formation was obtained by calculating rate constants for each elementary step and simulating the reaction progress at 300 and 450 °C, and they suggested that acetaldehyde is almost exclusively produced on both surfaces. These theoretical results were consistent with their temperature-programmed reaction experiments under a steady reactant feed up to ∼390 °C, including ethanol and O_2_, performed by Li et al. employing ceria nanoshapes.^22^ However, Li et al.^22^ also observed ethylene release from their ceria nanoshapes during the decomposition of ethanol in the absence of O_2_, which is congruent with our TPSR-MS results on both CeO_2_ nanooctahedra and nanocubes. A possible explanation for the disagreement between the nature of the products of the ethanol decomposition suggested by the studies on the extended ceria surfaces as compared to those employing ceria nanoshapes could be related to the nature of the surface species that were used in their DFT calculations of the minimum energy paths in the reaction of ethoxy species, as well as the considered intermediates. In the case of the (111) surface, the monodentate standing up ethoxy (monodentate type I in our case) had been considered as the initial state, whereas in our work, it is proposed that ethylene is produced from monodentate type II ethoxy (that is, lying down conformation) on CeO_2_-NO. Moreover, ethylenedioxy species, OCH_2_CH_2_O_latt_, are observed when ethylene is formed, which had not been considered in their calculations. Thus, the ethoxy and ethylenedioxy intermediate proposed here for the production of ethylene can account for our experimental results and also the results of Li et al.^22^ for ethoxy decomposition on (111) and (100) facets of ceria nanooctahedra and nanocubes.

Furthermore, if we compare our result for the ethylene formation for the example of the hydroxylated CeO_2_(111) surface (C_2_H_5_O_m-II_ + 2 O_latt_H + OH → CH_2_CH_2_ + H_2_O + OH + O_latt_H + O_latt_) with an energy barrier of 1 eV (Figure 8) with those reported by Beste et al.^68^ and recently by Salcedo et al.^78^ (C_2_H_5_O + O_latt_H → CH_2_CH_2_+ O_latt_H + OH) for the non-hydroxylated surface with barriers of 2.08 and 1.19 eV, respectively, we conclude that the presence of hydroxyl groups that give rise to the formation of water with OH species after the abstraction of the β-H from the C_2_H_5_O_m-II_ species plays a fundamental role in the reduction of the activation energy barrier for the formation of ethylene from C_2_H_5_O + O_latt_H up to 20% (we compare 1 and 1.19 eV since the mechanisms only differ in the fate of the extracted β-H and the methodology used in this work and in ref (78) is the same, which is not the case for ref (68), where, in addition to the different methodology, the way in which the O–C bond breaks differs). In summary, considering that the hydroxylated surface reproduces the experimental conditions more faithfully, our theoretical results should be more reliable.

Finally, the experimentally detected ethylenedioxy species, the binding of which depends on the orientation of the exposed ceria facets, do not only explain the larger amounts of gas-phase ethylene produced on the (111) facets, on which the intermediates are much less strongly bound as compared to the (100) facets, but also the observed enhanced yield toward hydrogen production for the nanocubes. The more strongly bound intermediate on the (100) facets results in an increased probability of formation of hydrogen via formate species with the participation of the more labile lattice oxygen atoms on this facet.

## 5 Conclusions

The interaction of ethanol over ceria nanooctahedra and nanocubes, which mainly expose (111) and (100) facets, respectively, as proven by TEM, was studied by means of mass spectrometry, infrared spectroscopy, and DFT calculations. The temperature-programmed surface reaction of ethanol produces H_2_, C_2_H_4_, CO_2_, and CH_4_ over both ceria surfaces. Although H_2_ and ethylene had a similar evolution on the ceria nanooctahedra, showing a maximum at 268 °C, on the nanocubes, ethylene was produced at higher temperatures as compared to H_2_ (maximum at 290 and 264 °C, respectively). Additionally, the ethylene peak was twice as large on the octahedra as in the cubes, while higher amounts of H_2_, CO_2_, and CH_4_ could be observed on the cubes with respect to the octahedra.

The correlation of the results of TPSR-MS and IR in transmittance mode together with DFT calculations allowed us to propose a mechanism for the reaction of ethanol on the surfaces of the ceria nanoshapes explored in this work, allowing us to explain the shape-dependent differences found in the TPSR-MS.

In the case of CeO_2_ nanooctahedra, after ethanol adsorption, monodentate type I and II ethoxy species were formed on the surface, with the alkyl chain parallel or perpendicular to the surface, respectively. Monodentate type I ethoxy species, which is the less stable surface species, decomposed to acetate species that result in CO_2_ and CH_4_ in the gas phase, leaving an oxygen vacancy on the surface. On the other hand, the more stable monodentate type II ethoxy species decomposes to give a labile ethylenedioxy intermediate (OCH_2_CH_2_O_latt_), which desorbed as ethylene in the gas phase.

On the case of ceria nanocubes, two types of ethoxy species were also detected. By means of DFT calculations, the ethoxy species were identified as bidentate ethoxy species on the checkerboard O-terminated (100) surface and a monodentate type III ethoxy species on a pyramid of the (100) surface that has a mixture of the O- and Ce- terminations (75% [(100)-O]-25% [(100)-Ce]). As in the case of the nanooctahedra, the more labile monodentate type III ethoxy species decomposes into acetate species that result in CO_2_, CH_4_, and an oxygen vacancy. On the other hand, bidentate ethoxy species were proposed as responsible for the formation of a very stable ethylenedioxy intermediate, which partially desorbed as ethylene but further reacted to give formate species via C–C cleavage. That formate species, not detected on the nanooctahedra, decomposes to H_2_ and CO_2_, leaving two oxygen vacancies on the surface. Thus, both ethoxy species on the (100) facets contribute to CO_2_ production. Furthermore, the molecular hydrogen evolution that results from the recombination of H and/or OH species has been found to be similar on both types of surfaces. However, the higher H_2_ released in the gas phase in the case of CeO_2_ nanocubes has been explained by an additional pathway, that is, the decomposition of the strongly bound ethylenedioxy intermediate on the (100) facets.

Finally, under ESR conditions, the enhanced reactivity observed for ceria nanooctahedra as compared to the nanocubes is due to the increased dehydration probability of ethanol to ethylene on the (111) facets, in line with the results of the TPSR experiments. In contrast, the nanocubes are more efficient for breaking the C–C bond of a tightly adsorbed ethylenedioxy intermediate on the (100) facets, resulting in a higher yield toward H_2_.

## Supporting Information Available

Definition of catalytic performance parameters, additional details on the calculations of ethoxy, TPSR-IR of CH_3_CD_2_OH, calculated IR spectra of ethylenedioxy species, integrated intensity of acetate and Ce^3+^ IR bands during TPSR-IR normalized by the surface are of each sample, catalytic evaluation results (PDF)

## Notes

The DFT data that support the findings of this study are available in Materials Cloud {https://www.materialscloud.org/home} with the identifier doi: 10.24435/materialscloud:1p-7e.
