# Supplementary material for: Shape-Controlled Pathways in the Hydrogen Production from Ethanol Steam Reforming over Ceria Nanoparticles
Source: ACS Catal. 2022 Aug 10;12(16):10482–98. doi: 10.1021/acscatal.2c02117 (PMC9396663; doi:10.1021/acscatal.2c02117)
Supplement: Supplementary file 1 — cs2c02117_si_001.pdf [file cs2c02117_si_001.pdf]

Supporting Information

Shape-controlled pathways in the hydrogen production from ethanol steam reforming over ceria nanoparticles

Julia Vecchietti,[1] Patricia Pérez-Bailac,[2,3] Pablo G. Lustemberg,*[2,4] Esteban L. Fornero,[1] Laura Pascual,[2] Marta V. Bosco,[1] Arturo Martínez-Arias,[2] M. Verónica Ganduglia-Pirovano,[2] Adrian L. Bonivardi[1,5]*

[1] Instituto de Desarrollo Tecnológico para la Industria Química, UNL-CONICET, Güemes 3450, 3000 Santa Fe, Argentina.

[2] Instituto de Catálisis y Petroleoquímica, CSIC, C/Marie Curie 2, 28049 Madrid, Spain

[3] PhD Programme in Applied Chemistry, Doctoral School, Universidad Autónoma de Madrid, C/Francisco Tomas y Valiente 2, 28049 Madrid, Spain

[4] Instituto de Fisica Rosario (IFIR), CONICET-UNR, Bv. 27 de Febrero 210bis, 2000EZP Rosario, Santa Fe, Argentina

[5] Facultad de Ingeniería Química, Universidad Nacional del Litoral, Santiago del Estero 2829, 3000 Santa Fe, Argentina.

Corresponding Author

*Email: jvecchietti@santafe-conicet.gov.ar

*Email: p.lustemberg@csic.es

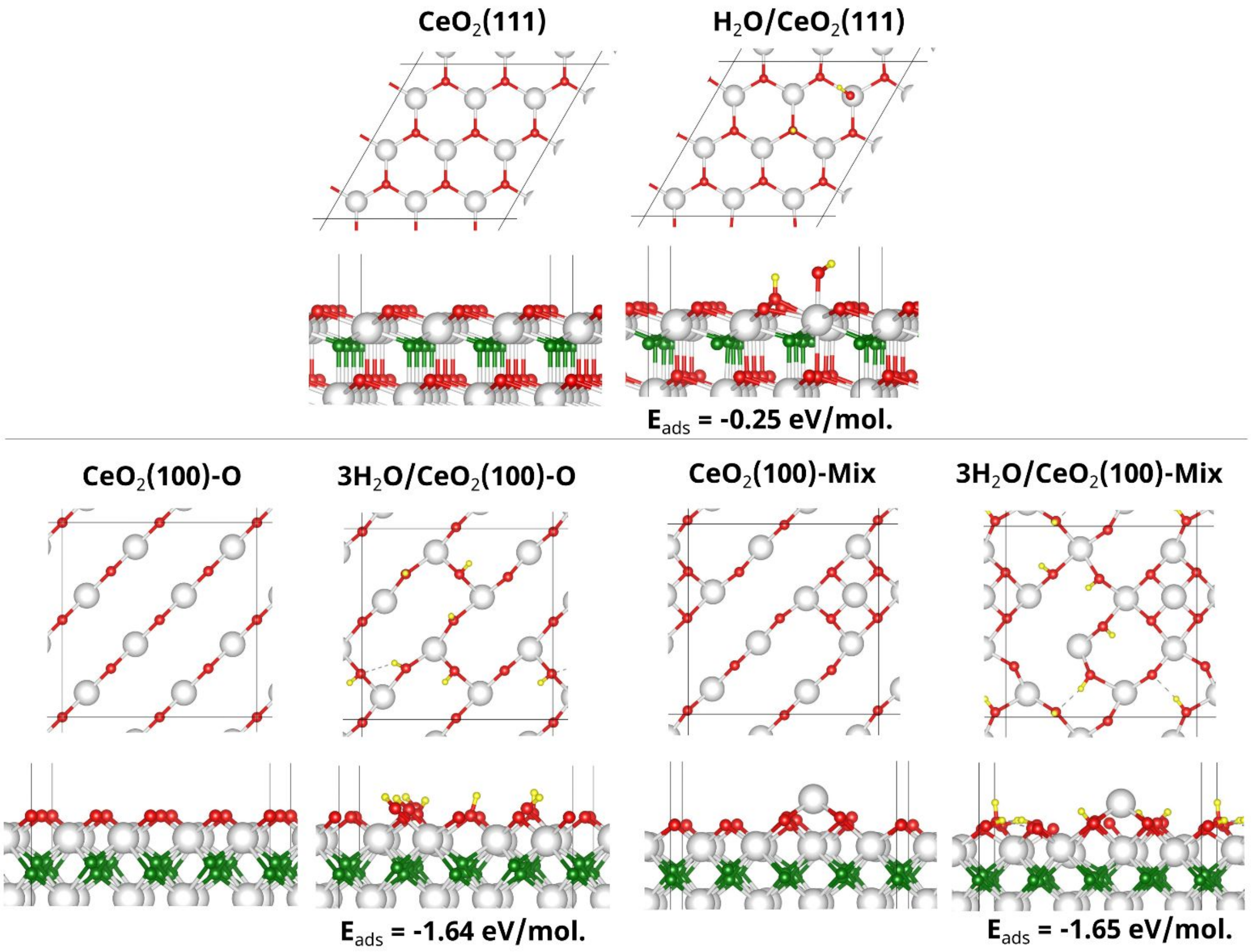

Figure S1. Top and side view of the bare and hydroxylated CeO_2 (111), (100)-O and (100)-Mix surfaces. The adsorption energy per water molecule is reported.

Scaling of theoretical frequencies

The theoretical frequencies were scaled following the procedure described by Merrick et al.[1] minimizing by least-squares the residual:

$$\Delta = \sum_i^{all}\left(\lambda\omega_i^{theor} - \nu_i^{exp}\right)^2 \qquad \text{(S1)}$$

where ω_i^{theor} and ν_i^{exp} are the *i*th theoretical harmonic frequency and the *i*th experimental frequency (in cm^{-1}) of ethanol in gas-phase in the selected range. The experimental data were obtained from the NIST database (https://cccbdb.nist.gov/). The minimization of equation (1) leads to:

$$\lambda = \frac{\sum_i^{all}\omega_i^{theor}\nu_i^{exp}}{\sum_i^{all}\left(\omega_i^{theor}\right)^2} \qquad \text{(S2)}$$

The optimized scale factor λ was then used to calculate an overall root-mean-square error, rms_{ov}:

$$rms_{ov} = \left(\sum_{1}^{n_{mol}} \frac{\Delta_{min}}{n_{mol}}\right)^{\frac{1}{2}} \qquad \text{(S3)}$$

where n_{mol} is the number of modes and $\Delta_{min} = \left(\lambda\omega_i^{theor} - \nu_i^{exp}\right)^2$. With this procedure the scale factor obtained and applied to the DFT frequencies values was λ = 1.019 with rms_{ov} = 4 cm^{-1} in the 800 – 1200 cm^{-1} range and λ = 0.987 with rms_{ov} = 4 cm^{-1} in the 2800 – 3200 cm^{-1} (cf. Table S1 for experimental and calculated frequencies for ethanol in the gas phase).

Table S1: Experimental and calculated frequencies of ethanol in gas phase in the 800−1200 cm^{-1} range. These values were used to find the scale factor λ.

Vibrational mode	Experimental (cm^{-1})	Calculated (cm^{-1})	λ
$\nu_{as}(CH_3)$	3056	2984	0.987
$\nu_s(CH_3)$	2981	2939	
$\nu_{as}(CH_2)$	2938	2910	
$\nu_s(CH_2)$	2913	2900	
τ(CCO)	1161	1135	1.019
ν(CO)	1091	1068	
ν_{as}(CCO)	1028	1007	
ν_s(CCO)	888	878	

Conversion and yield definitions to evaluate the catalytic performance

Conversion of ethanol ($X_{C_2H_5OH}$), %:

$$X_{C_2H_5OH} = \frac{(m_{C_2H_5OH}^{in} - m_{C_2H_5OH}^{out})}{m_{C_2H_5OH}^{in}} * 100$$

Yield to hydrogen ($Yield_{H_2}$), %:

$$Yield_{H_2} = \frac{m_{H_2}^{out}}{6 * \ m_{C_2H_5OH}^{in}} * 100$$

Yield to carbon-containing compounds ($Yield_i$), %:

$$Yield_i = \frac{n_{C_i} * m_i^{out}}{2 * m_{C_2H_5OH}^{in}} * 100$$

$m_{C_2H_5OH}^{in}$: moles of ethanol at the inlet of the reactor

$m_{C_2H_5OH}^{out}$: moles of ethanol at the reactor outlet

$m_{H_2}^{out}$: moles of H_2 at the reactor outlet

n_{C_i}: number of carbon atoms of the *i*-product

m_i^{out}: moles of the *i*-product at the reactor outlet

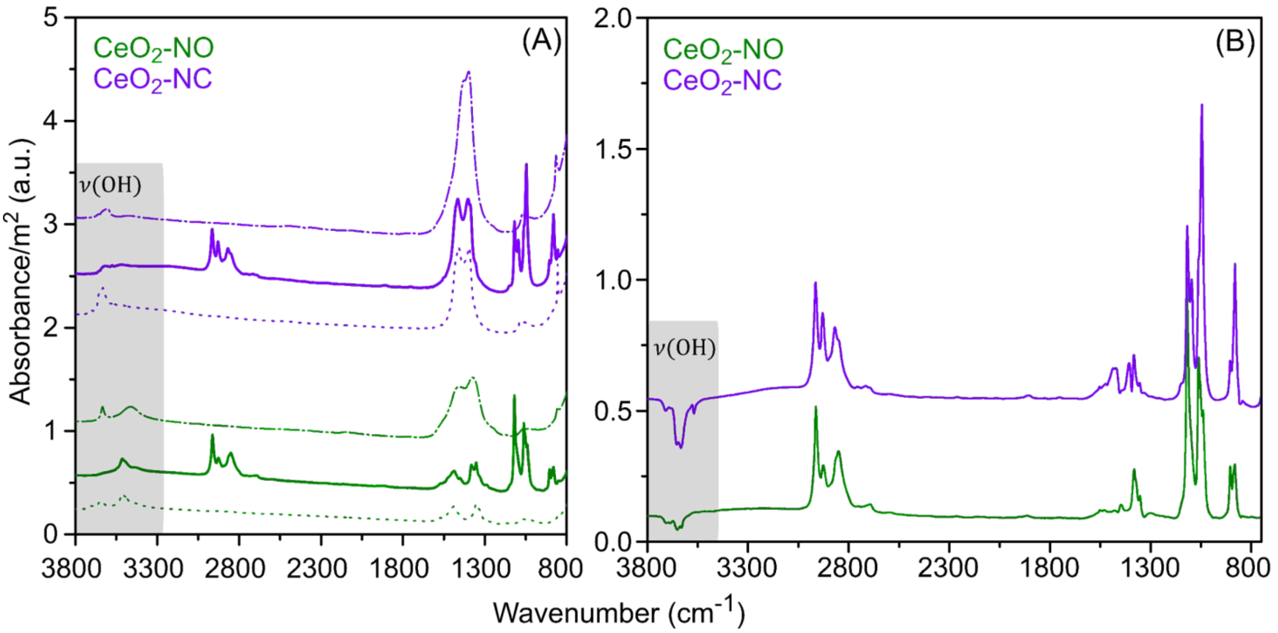

Figure S2. IR spectra for CeO_2-NO and CeO_2-NC (A) after pretreatment (dotted lines), ethanol adsorption at 100 °C and purging with He (full lines) and after TPSR at 450°C (dash-dot lines), and (B) ethanol adsorption and purging with He after subtraction of the spectrum of the clean oxide right before the adsorption (full line minus dotted line spectra).

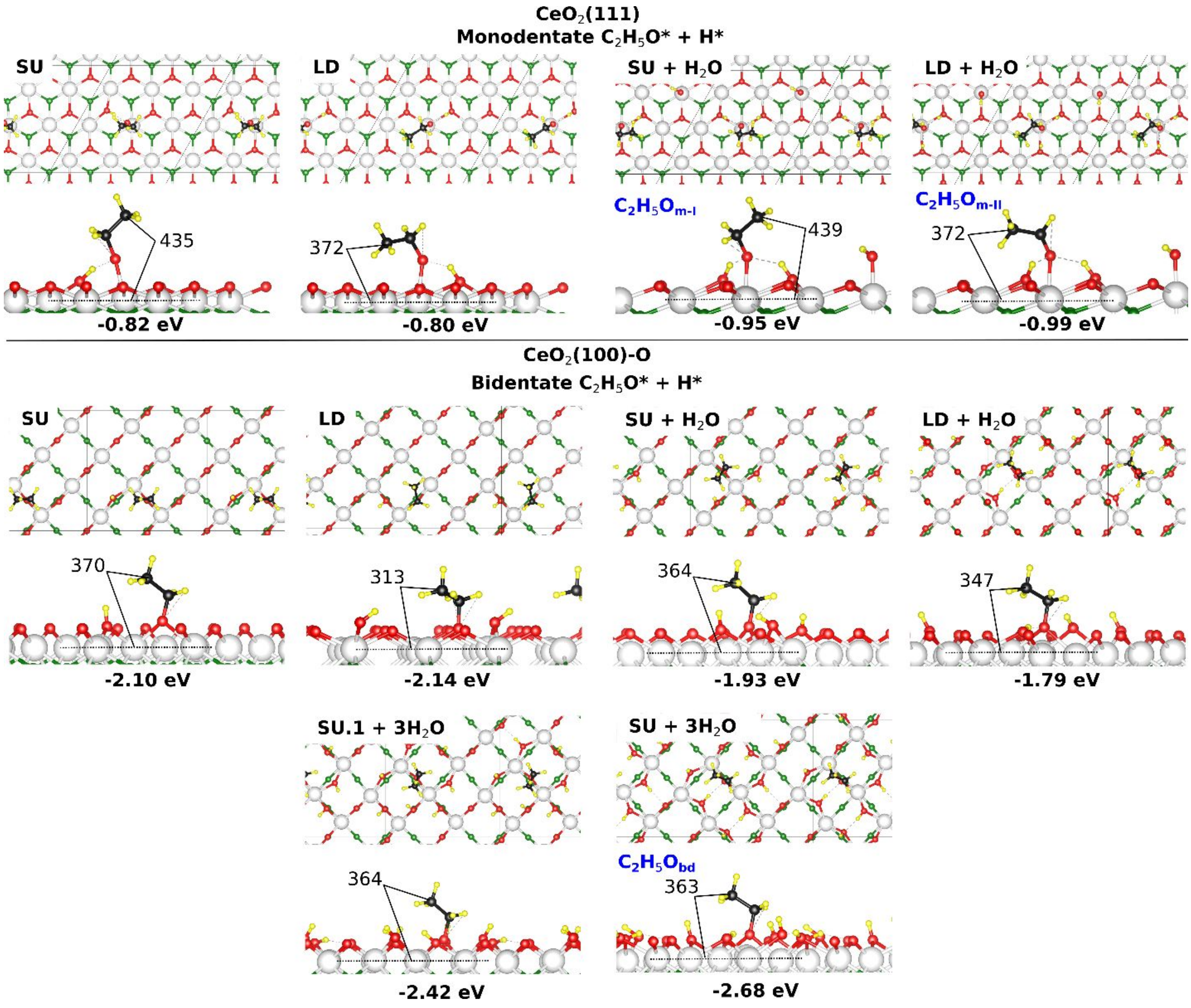

Figure S3. Top and side views of the dissociative adsorption of ethanol on ceria surfaces. Monodentate standing-up, SU, and lying-down, LD, ethoxy species on non-hydroxylated and hydroxylated (1 H_2O) $CeO_2(111)$ surfaces are shown, according to Ref. 2. On $CeO_2(100)$-O with up to 3 H_2O water molecules, bidentate ethoxy SU and LD are shown. The adsorption energy with respect to the (hydroxylated) ceria surface and gas phase ethanol in eV and the closest distance from the C2 atom to a surface Ce in pm are indicated. The SU+1H_2O and LD+1H_2O on $CeO_2(111)$ correspond to the monodentate type I and II ($C_2H_5O_{m\text{-}I}$ and $C_2H_5O_{m\text{-}II}$) species in the main text and SU+3H_2O on $CeO_2(100)$ to the bidentate ($C_2H_5O_{bd}$).

Two types of ethoxy species, LD and SU, are observed in the non-hydroxylated $CeO_2(100)$, as in the case of the $CeO_2(111)$. Unlike the (111) facet, as the degree of hydroxylation increases, the *lying down* (LD) state becomes *standing up* (SU.1+3H_2O), which is 0.26 eV less stable than SU.3H_2O, which in the main text notation is $C_2H_5O_{bd}$, and whose frequency difference is less than 6 cm^{-1} (see Table S2). These results indicate that only one type of ethoxy species would exist in the $CeO_2(100)$-O with checkerboard termination.

Table S2: Calculated scaled frequencies of standing-up, SU, and lying-down, LD, ethoxy species on CeO_2(100)-O, with 0, 1 and 3 dissociated water molecules (cf. Figure S2). The adsorption energy and the frequency shift, $\Delta\nu = \nu(LD) - \nu(SU)$ are given in eV and cm^{-1}, respectively. Note that in the case of the coadsorption of ethoxy species with 3 water molecules, there is not a lying-down species.

Vibrational mode	LD	SU	Δv/ΔE	LD+H_2O	SU+H_2O	Δv/ΔE	SU+3H_2O	SU.1+3H_2O	Δv/ΔE
ν(CO)	1096	1090	+6	1101	1088	+13	1096	1090	+6
ν_{as}(CCO)	1032	1047	-15	1033	1043	-10	1041	1039	+2
ν_s(CCO)	886	894	+2	876	887	-11	883	881	+2
**Energy**	**-2.14**	**-2.10**	**-0.04**	**-1.79**	**-1.93**	**+0.14**	**-2.68**	**-2.42**	**-0.26**

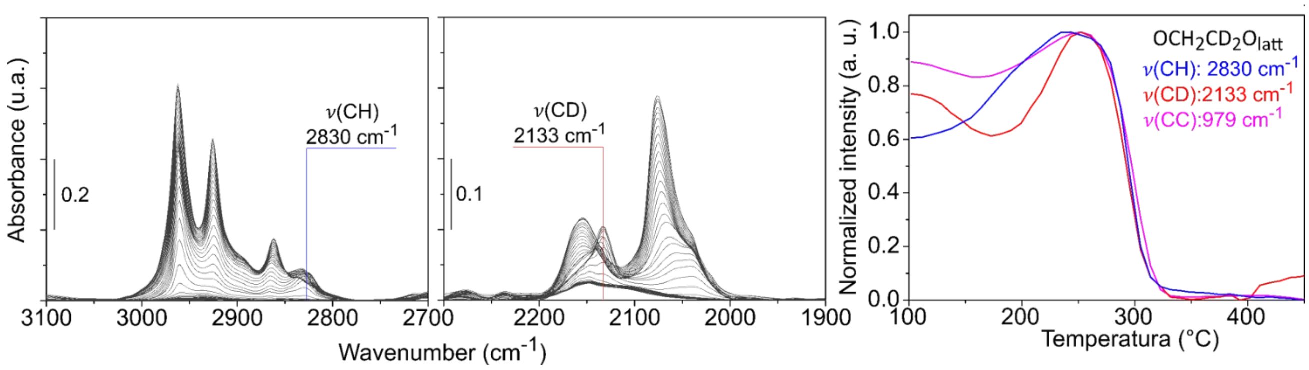

Figure S4. TPSR-IR of CH_3CD_2OH for CeO_2-NO.

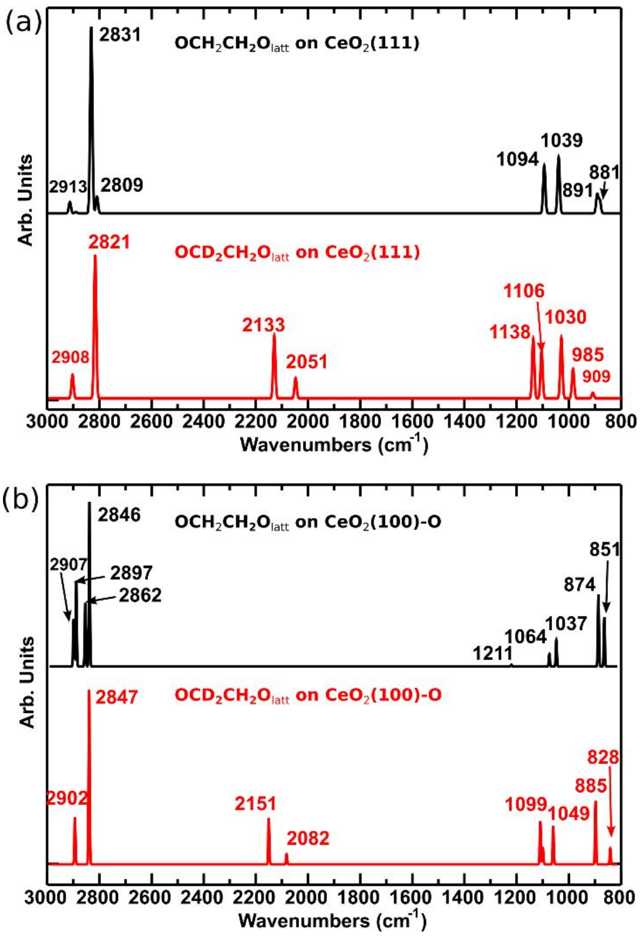

Figure S5. Calculated IR spectra of $OCH_2CH_2O_{latt}$/$OCH_2CD_2O_{latt}$ adsorbed on a) $CeO_2(111)$ plus 1 dissociated H_2O and b) $CeO_2(100)$-O plus 3 dissociated H_2O.

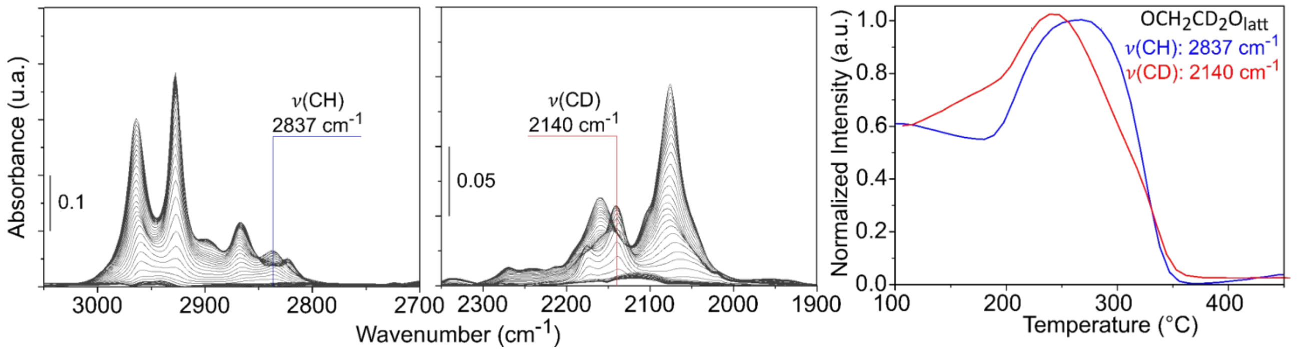

Figure S6 TPSR-IR of CH_3CD_2OH for CeO_2-NC.

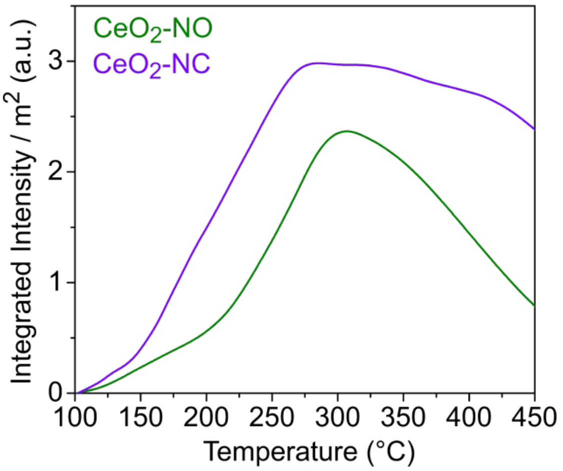

Figure S7. Integrated Intensity of acetate species during the TPSR-IR of ethanol for CeO_2-NO and CeO_2-NC. Integration of the ~1425 cm^{-1} signal, that is, the most intense and less overlapped, was performed after subtraction of the spectra after ethanol adsorption and purging with He, right before the begging of the TPSR experiment. The evolutions are normalized by surface area of each ceria wafer.

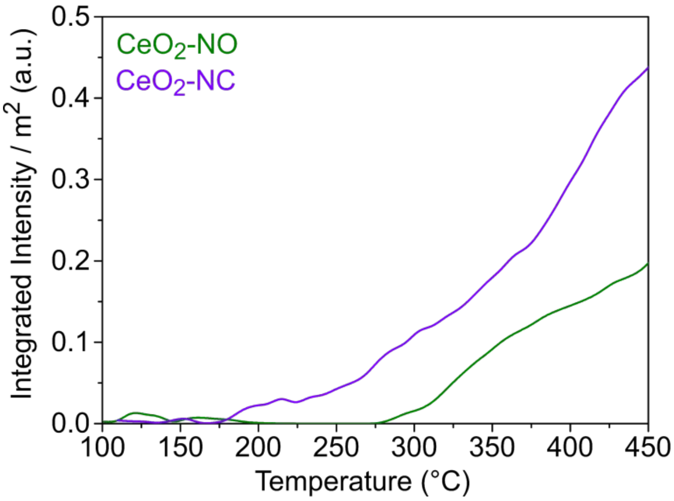

Figure S8. Integrated Intensity of Ce^{3+} species (2130 cm^{-1} signal) during the TPSR-IR of ethanol for CeO_2-NO and CeO_2-NC. The evolutions are normalized by surface area of each ceria wafer.

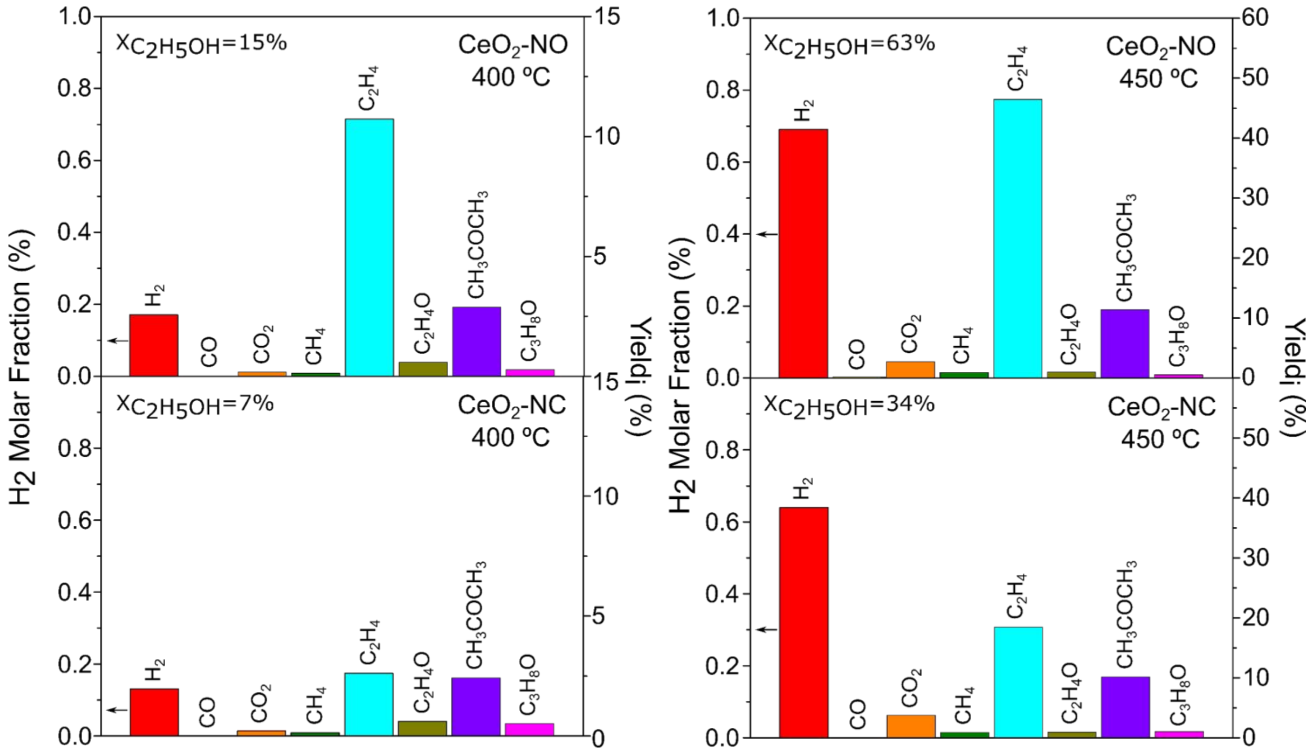

Figure S9. Product yield at 400 and 450 °C under ESR conditions (H_2O:C_2H_5OH=6:1, 800 m^2.h/molC_2H_5OH) for CeO_2-NC and CeO_2-NC. X_{C2H5OH} stands for conversion of ethanol.

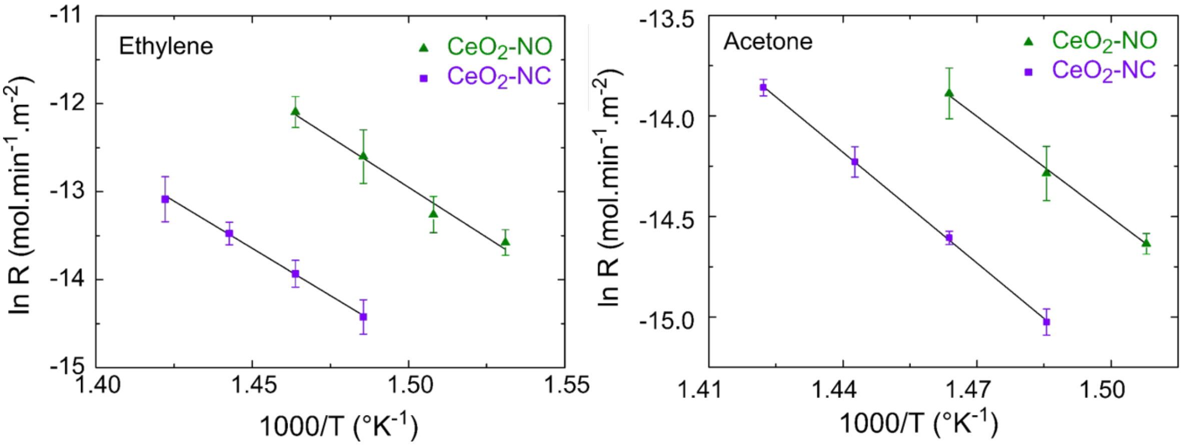

Figure S10. Arrhenius plot for acetone and ethylene production over CeO_2-NO and CeO_2-NC samples. Reaction conditions: H_2O/C_2H5OH = 6/1 mol/mol; T = 380-430 °C; W/F = 530 m^2.h/mol_{C2H5OH} for NC and 274 m^2.h/mol_{C2H5OH} for NO.

References

(1) Merrick, J. P.; Moran, D.; Radom, L. An Evaluation of Harmonic Vibrational Frequency Scale Factors. *J. Phys. Chem. A* **2007**, *111* (45), 11683–11700.

(2) Vecchietti, J.; Lustemberg, P.; Fornero, E. L.; Calatayud, M.; Collins, S. E.; Mohr, S.; Ganduglia-Pirovano, M. V.; Libuda, J.; Bonivardi, A. L. Controlled Selectivity for Ethanol Steam Reforming Reaction over Doped CeO_2 Surfaces: The Role of Gallium. *Appl. Catal. B Environ.* **2020**, *277*, 119103. https://doi.org/10.1016/j.apcatb.2020.119103.
